# Antithrombotic therapy in patients with liver disease: population-based insights on variations in prescribing trends, adherence, persistence and impact on stroke and bleeding

**DOI:** 10.1016/j.lanepe.2021.100222

**Published:** 2021-09-08

**Authors:** Wai Hoong Chang, Stefanie H. Mueller, Yen Yi Tan, Alvina G. Lai

**Affiliations:** a Institute of Health Informatics, University College London, London, UK

**Keywords:** Antiplatelets, anticoagulants, liver disease, adherence, persistence, prescribing pattern, stroke, bleeding

## Abstract

**Background:**

Patients with liver disease have complex haemostasis and due to such contraindications, landmark randomised controlled trials investigating antithrombotic medicines have often excluded these patients. As a result, there has been limited consensus on the safety, efficacy and monitoring practices of anticoagulant and antiplatelet therapy in patients with liver disease. This study aims to investigate prescribing prevalence, adherence, persistence and impact of adherence on bleeding and stroke risk in people with and without liver disease taking anticoagulants and antiplatelets.

**Methods:**

We employed a population-based cohort consisting of person-level linked records from primary care, secondary care and the death registry. The cohort consisted of 3,929,596 adults aged ≥ 30 years during the study period of 1998 to 2020 and registered with an NHS general practitioner in England. The primary outcome was prescribing prevalence, adherence to and persistence with anticoagulant and antiplatelet therapy comparing patients with and without liver disease. Risk factors for non-adherence and non-persistence were analysed using multivariable logistic regression and Cox regression. Impact of adherence on bleeding and ischaemic stroke was assessed.

**Findings:**

Among patients with any of the six liver diseases (ALD, autoimmune liver disease, cirrhosis, HBV, HCV and NAFLD), we identified 4,237 individuals with incident atrial fibrillation (indication for anticoagulants) and 4,929 individuals with incident myocardial infarction, transient ischaemic attack, unstable angina or peripheral arterial disease (indication for antiplatelets). Among patients without liver disease, 321,510 and 386,643 individuals were identified as having indications for anticoagulant and antiplatelet therapy, respectively. Among drug-naïve individuals, prescribing prevalence was lower in patients with liver disease compared with individuals without liver disease: anticoagulants (20.6% [806/3,921] vs. 33.5% [103,222/307,877]) and antiplatelets (56.2% [2,207/3,927] vs. 71.1% [249,258/350,803]). Primary non-adherence rates (stopping after one prescription) were higher in patients with liver disease, compared with those without liver disease: anticoagulants (7.9% [64/806] vs. 4.7% [4,841/103,222]) and antiplatelets (6.2% [137/2,207] vs. 4.4% [10,993/249,258]). Among individuals who were not primary non-adherent and had at least 12 months of follow-up, patients with liver disease however had a higher one-year adherence rate: anticoagulants (33.1% [208/628] vs. 29.4% [26,615/90,569]) and antiplatelets (40.9% [743/1,818] vs. 34.4% [76,834/223,154]). Likelihood of non-adherence was lower in apixaban and rivaroxaban (relative to warfarin) and lower in clopidogrel (relative to aspirin). Increased comorbidity burden (by CHA_2_DS_2_VASc score) was associated with decreased risk of non-adherence and non-persistence with anticoagulants. Overall rates of ‘non-adherent, non-persistent’ were highest in warfarin (compared with apixaban and rivaroxaban) and aspirin (compared with clopidogrel or dipyridamole) in patients with and without liver disease. Among patients without liver disease, not taking antithrombotic medications for >3 months was associated with a higher risk of stroke, however, adherence to these medications was also associated with a small increase in risk of bleeding. Patients with liver disease (when compared with those without liver disease) had higher risks of stroke, especially when they stopped taking antiplatelets for >3 months. Patients with liver disease who were adherent to antiplatelets, however, had a higher risk of bleeding compared with patients without liver disease.

**Interpretation:**

Use of antithrombotic medicines in patients with and without liver disease is suboptimal with heterogeneity across medicines. As patients with liver disease are excluded from major randomised trials for these drugs, our results provide real-world evidence that may inform medicine optimisation strategies. We outline challenges and opportunities for tackling non-adherence, which begins with understanding patients’ views of medicines to help them make informed decisions about appropriate use.

**Funding:**

AGL is supported by funding from the Wellcome Trust (204841/Z/16/Z), National Institute for Health Research (NIHR) University College London Hospitals Biomedical Research Centre (BRC714/HI/RW/101440), NIHR Great Ormond Street Hospital Biomedical Research Centre (19RX02), the Health Data Research UK Better Care Catalyst Award (CFC0125) and the Academy of Medical Sciences (SBF006\1084). The funders have no role in the writing of the manuscript or the decision to submit it for publication.

**Research in context**

**Evidence before this study**

Evidence on the use of antithrombotic medications in patients with liver disease has been inconclusive as these individuals are excluded from major randomised trials testing the efficacy and safety of these drugs. We searched PubMed from database inception to 30 May 2021 for studies reporting antithrombotic use in patients with liver disease using the following search terms: “chronic liver disease”, “cirrhosis”, “non-alcoholic fatty liver disease”, “hepatitis B, “hepatitis C”, “autoimmune liver disease”, “alcoholic liver disease”, “antithrombotic”, “antiplatelet”, “anticoagulant”, “prescribing pattern”, “prescribing prevalence”, “adherence”, “persistence”, “bleeding” and “stroke”. We found that although some observational studies have demonstrated that patients with liver disease and atrial fibrillation may benefit from anticoagulant use for stroke prevention, results have been inconsistent. Furthermore, most studies have focused on cirrhosis or non-alcoholic fatty liver disease – other less common forms of liver diseases have not been investigated. We did not identify any study investigating the relationship between adherence and persistence patterns (for anticoagulants and antiplatelets) and risk of stroke or bleeding, and comparing results between patients with and without liver disease.t

**Added value of this study**

This is the first study investigating prescribing prevalence, adherence, persistence, interaction between adherence and persistence, and impact of adherence on stroke and bleeding risks for both anticoagulants and antiplatelets in patients with and without liver disease using a single nationwide cohort. We found that prescribing prevalence of anticoagulants and antiplatelets was lower in patients with liver disease compared with individuals without liver disease. Heterogeneity in prescribing prevalence, adherence to and persistence with antithrombotic medicines was observed across different drug types and geographical regions in England. Patients with liver disease had higher rates of primary non-adherence. Nonetheless, among individuals who had more than one prescription (not primary non-adherent), adherence was higher in patients with liver disease (compared with people without liver disease). Non-adherence to antiplatelets for longer than 3 months in patients with liver disease was associated with increased stroke risk (compared with people without liver disease). However, adherence to antiplatelets in patients with liver disease was associated with increased bleeding risk.

**Implications of all available evidence**

Our work has significant implications on 1) identification of potentially unwarranted regional variations in antithrombotic prescribing, adherence and persistence, 2) identification of high-risk individuals for risk-benefit assessments for the management of antithrombotic therapy in liver disease, 3) involving patients in shared decision-making in the choice and duration of therapy while minimising non-adherence and 4) additional monitoring procedures for patients with liver disease that involves screening for ongoing alcohol use, assessing liver function and measuring coagulation profile before and during therapy.

Alt-text: Unlabelled box

## 1 Introduction

The prevalence of liver disease is steadily rising over the years due to the increasing prevalence of conditions with similar risk factors (e.g., diabetes mellitus, obesity and dyslipidaemia) [1]. Liver disease has an insidious onset; progressing gradually over years or even decades with people experiencing limited and intermittent clinical signs in early stages of the disease. Non-alcoholic fatty liver disease (NAFLD) affects a staggering number of two billion [2] individuals worldwide with a disease burden that is expected to increase in the coming years [3]. Despite these issues, public health policies on liver disease prevention have been lukewarm. A survey on 29 European countries demonstrated that none of these countries have action plans for tackling NAFLD, and awareness campaigns are lacking [4].

Over time, due to shared risk factors, many people with liver disease go on to develop cardiovascular disease (CVD) [5,6], necessitating the use of drugs to prevent the exacerbation of thrombotic events [7]. There has also been limited progress on public health policies targeting people with both liver disease and CVD, causing significant life and economic losses. Patients with advanced liver disease not only suffer from prothrombotic disorders but also have an increased risk of bleeding [8]. As liver disease affects the hepatic clearance of many drugs, altered levels of liver enzymes may affect response to medicines and cause liver injury [9]. Despite the need for more evidence on drug safety and efficacy, individuals with active liver disease were excluded from landmark direct oral anticoagulant (DOAC) trials [10], [11], [12], [13]. Because of limited trial evidence on DOACs, warfarin (a vitamin K antagonist) is a common choice for people with liver disease. Nonetheless, the use of warfarin in patients with liver disease and active coagulopathy poses additional challenges in the selection of suitable dosing due to deranged INR values [14]. Given their availability, DOACs have been used in clinical practice in patients with mild liver disease. The European Heart Rhythm Association guideline mentioned that all four DOACs are contraindicated in patients with liver disease especially in patients with Child-Pugh class C [15]. The guideline recommends that rivaroxaban should not be used in patients with Child-Pugh class B cirrhosis due to a 2-fold increase in drug exposure in these patients [16], however, dabigatran, apixaban and edoxaban may be used with caution. A meta-analysis evaluating bleeding and thromboembolic complications in patients with alcoholic liver disease who were prescribed with DOACs or warfarin found that the safety and efficacy of DOACs in patients with alcoholic liver disease were not significantly different from those without liver disease [17]. The European Heart Rhythm Association guideline recommends that the initiation and follow-up of anticoagulant therapy should include multidisciplinary teams involving hepatologists and haematologists [15]. As hepatic impairment could affect the metabolism of these drugs, the European Medicines Agency (EMA) [18] recommends additional pharmacokinetic studies in patients with impaired liver function and assessing the degree of impairment (i.e., the presence of ascites, varices and encephalopathy) as part of disease management.

Antiplatelets are employed to reduce the risk of stroke after acute coronary syndrome and to prevent future atherothrombotic events in peripheral arterial disease. Interestingly, antiplatelet therapy has been shown to be inversely associated with the progression of liver fibrosis. Others have demonstrated significant correlation between platelet levels and concentration of PDGF-β (a driver of liver fibrosis), however, antiplatelet therapy did not affect PDGF-β levels despite exhibiting protective effects on liver fibrosis [19]. Since platelets are essential to maintaining haemostasis, a fine balance between the risk of bleeding and prevention of future cardiovascular events must be kept. P2Y12 receptor antagonists, such as clopidogrel, ticagrelor and prasugrel, are popular antiplatelet medicines as they appear to provide thrombotic protection with limited risk of bleeding. Clopidogrel and prasugrel must be converted into active metabolites in the liver before they can bind to the platelet P2Y12 receptor to confer antiplatelet effects [20]. Therefore, caution is recommended in people with hepatic impairment [21]. However, since landmark trials for clopidogrel (i.e., CLARITY and COMMIT) have excluded patients with hepatic insufficiency [22], there is limited evidence on safety and efficacy in such patients. Similarly, the prasugrel trial excluded people with liver disease (especially cirrhosis), people with a history of alcoholism and those who are at increased risk of bleeding [23].

Restrictive eligibility criteria in antithrombotic trials have resulted in limited generalisability of results to people with liver disease. With the rising prevalence of atrial fibrillation and coronary heart disease in these individuals, as well as growing treatment options, insights from electronic health records can provide a much-needed evidence-base on antithrombotic use, patterns of adherence (taking medication as prescribed) and persistence (treatment continuation) and safety and efficacy profiles in these patients. Using primary and secondary care population health records from 4 million individuals, the objectives of our study are: 1) to investigate geographical variations in prescribing prevalence of five anticoagulants and five antiplatelet medications in people with and without liver disease, 2) to estimate adherence to and persistence with anticoagulants and antiplatelets (at 6 and 12 months) in people with and without liver disease, and differences across geographical regions, 3) to explore clinical factors associated with the risk of non-adherence and non-persistence (at 6 and 12 months), 4) to investigate the interactions between adherence and persistence, 5) to investigate the impact of adherence on bleeding risk and 6) to investigate the impact of non-adherence (short or long-term discontinuation of therapy) on risk of ischaemic stroke. Coordinated efforts across cardiology and hepatology specialties and multidisciplinary teams are required to improve our understanding of how antithrombotic therapy can be managed optimally. In patients with liver disease who are often contraindicated, addressing non-adherence may require overcoming specialty silos and involving patients in shared decision making.

## 2 Methods

### 2.1 Dataset and electronic health record phenotypes

Electronic health records in a cohort of 3,929,596 adults aged ≥ 30 years during the study period of 1998 to 2020 from primary care linked to secondary care and the death registry were analysed. Follow-up ceased at the occurrence of a primary endpoint, death, date of last data collection for the practice, date of administrative censoring (June 2020) or deregistration from the practice (i.e., loss to follow-up), whichever occurred first. Information governance approval was obtained from the Medicines Healthcare Regulatory Authority (UK) Independent Scientific Advisory Committee (21_000363) Clinical Practice Research Datalink (CPRD).

Baseline characteristics at the time of first anticoagulant or antiplatelet prescription in people with or without liver disease were analysed. Phenotype definitions for liver disease, cardiovascular disease (CVD), antithrombotic medications and comorbidities are available at https://caliberresearch.org/portal and have previously been validated [24,25]. Phenotypes for primary care records were generated using Read clinical terminology (version 2). Phenotypes for secondary care records were generated using ICD-10 terms. We considered five types of anticoagulants (apixaban, dabigatran, edoxaban, rivaroxaban and warfarin) and five types of antiplatelets (aspirin, clopidogrel, dipyridamole, prasugrel and ticagrelor). For stratified analyses involving specific medications, we have only analysed drug types that had more than 100 individuals.

### 2.2 Prescribing prevalence

Prescribing prevalence was analysed separately in patients with and without liver disease. For analysis on patients with liver disease, we considered all individuals with a diagnosis of any of the following six conditions: alcoholic liver disease (ALD), autoimmune liver disease (autoimmune hepatitis and primary biliary cholangitis), cirrhosis, chronic hepatitis B infection (HBV), chronic hepatitis C infection (HCV) or non-alcoholic fatty liver disease (NAFLD). Among patients with liver disease, we next identified individuals with incident CVD, which was diagnosed after the diagnosis of liver disease, given that we were interested in assessing prescribing patterns in individuals with pre-existing liver disease who were newly diagnosed with CVD. We considered atrial fibrillation as the indicated condition for anticoagulant therapy. Myocardial infarction, peripheral arterial disease, unstable angina and transient ischaemic attack were considered as indicated conditions for antiplatelet therapy. All individuals with prevalent liver disease and incident CVD indications were considered as the denominator population. A separate cohort was generated that consisted of all individuals without liver disease. Among these individuals, everyone with an incident CVD diagnosis was included in the denominator population. The prevalence of antithrombotic medications prescribing was presented per 100 persons and 95% confidence intervals were calculated according to the central limit theorem for dichotomous outcome (i.e., being prescribed with medication or not).

### 2.3 Liver disease severity

We estimated Child-Pugh score and FIB-4 score for each patient as indicators of liver disease severity. We also considered the presence of varices, ascites and hepatic encephalopathy as markers of advanced liver disease. Child-Pugh score was estimated based on five clinical measures: ascites, hepatic encephalopathy, total bilirubin, serum albumin and International Normalised Ratio (INR). Patients were grouped into three Child-Pugh score classes: (i) Class A (well-compensated disease, score 5-6), (ii) Class B (significant functional compromise, score 7-9) and (iii) Class C (decompensated disease, score 10-15). FIB-4 score was estimated using four clinical measures: age, aspartate aminotransferase level, platelet count and alanine aminotransferase level. Patients were classified into 3 groups according to their FIB-4 scores: (i) <1.45 (approximate fibrosis stage 0-1), (ii) 1.45-3.25 (fibrosis stage 2-3) and (iii) > 3.25 (fibrosis stage 4-6).

### 2.4 Time in therapeutic range

For patients prescribed with warfarin, we estimated time in therapeutic range (TTR) using the Rosendaal method that relies on linear interpolation to assign an INR value to each day between two consecutive recorded INR values [26]. TTR was estimated as the percentage of time during which interpolated INR values fall between 2 and 3. TTR ranges between 0 to 100%. We first calculated the amount of the total shift in INR between two consecutive measures that is within therapeutic range (INR between 2 and 3). Then we calculate the percent of total shift and estimated the number of days since last visit that were within range.

### 2.5 Adherence and persistence

Patients with at least 6 or 12 months of follow-up were considered in adherence and persistence analyses at 6 or 12 months, respectively. This was to reduce potential bias in estimating adherence or persistence in short treatment periods. Patients with only one prescription (primary non-adherent) were not included in the analyses.

Following previously validated methodology [27], we estimated adherence as the proportion of days covered (PDC) over 6 months or 12 months after the first antithrombotic prescription. We assumed that each prescription would last for 30 days unless a new prescription was issued within 30 days, in which case the prescription's duration was assumed as the duration between the two prescriptions. Adherence was defined as PDC above 80% following previous studies [27,28].

Individuals were considered persistent until a prescription gap of 90 days was reached. Individuals who switched to an alternative medication within the same drug class (e.g., warfarin to rivaroxaban or clopidogrel to dipyridamole) were censored rather than considered non-persistent to the first medication prescribed. Persistence was estimated at 6 months and 12 months. Relative effects of drug type, age, sex and comorbidities on non-adherence and non-persistence were modelled using multivariable logistic regression and Cox proportional hazards regression, respectively. For multivariable analyses, models were fully adjusted for all other covariates considered. For Cox regression, we evaluated the proportional hazards assumption which was found to be met.

Data were analysed using R (3.6.3) with the following packages: AdhereR [29], survival, tidyverse, tableone, rgdal, broom, ggplot2 and ggmap.

## 3 Results

The study cohort included 3,929,596 individuals. We considered six liver diseases, i.e., ALD, autoimmune liver disease, cirrhosis, HBV, HCV and NAFLD. In patients with any of these liver conditions, we identified 4,237 individuals with incident atrial fibrillation (AF) – an indication for anticoagulant therapy. In individuals without liver disease, we identified 321,510 patients with incident AF (Figure S1). We considered incident myocardial infarction, transient ischaemic attack, unstable angina and peripheral arterial disease as indications for antiplatelet therapy. We identified 4,929 and 386,643 individuals as having conditions indicated for antiplatelet therapy in individuals with and without prevalent liver disease, respectively (Figure S1).

### 3.1 Patients with liver disease had a lower prescribing prevalence of antithrombotic medications compared with those without liver disease

Analyses on prescribing prevalence were performed on individuals with cardiovascular disease (CVD) indications for the respective drugs. We analysed prescribing prevalence for initial antithrombotic prescription in drug-naïve patients to minimise bias based on previous medicine use. After excluding non-drug naïve individuals, 3,921 (with liver disease) and 307,877 (without liver disease) individuals were included in the analysis on anticoagulant prescribing prevalence. For antiplatelet prescribing prevalence, 3,927 (with liver disease) and 350,803 (without liver disease) individuals were included (Figure S1).

**Figure 1:**
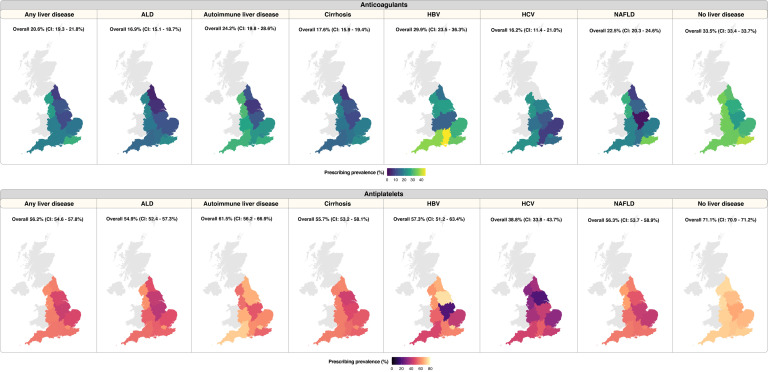
Prescribing prevalence of antithrombotic medications in individuals with cardiovascular indications. Prescribing prevalence was computed separately for patients with liver disease and those without liver disease. Cardiovascular indications were as follow: atrial fibrillation for anticoagulants; myocardial infarction, peripheral arterial disease, transient ischaemic attack, or unstable angina for antiplatelets. Overall prescribing prevalence for England is annotated above each map. CI: 95% confidence interval.

The prescribing prevalence of any anticoagulants (we have considered five anticoagulants: apixaban, dabigatran, edoxaban, rivaroxaban and warfarin) in patients with any of the six liver diseases was 20.6% [806/3,921] (95% confidence interval (CI): 19.3 – 21.8%). In contrast, prescribing prevalence of anticoagulants in people without liver disease was higher at 33.5% [103,222/307,877] (CI: 33.4 - 33.7%) (Figure 1, Table S3). When considering specific liver conditions, only 16.2% [37/228] (CI: 11.4 - 21.0%) of patients with HCV received anticoagulant prescriptions compared with 29.9% [58/194] (CI: 23.5 - 36.3%) of patients with HBV. Prescribing prevalence for anticoagulants in patients with other liver conditions were as follow: ALD (16.9% [275/1,629]; CI: 15.1 - 18.7%), cirrhosis (17.6% [322/1,827]; CI: 15.9 - 19.4%), autoimmune liver disease (24.2% [88/364]; CI: 19.8 - 28.6%) and NAFLD (22.5% [331/1,474]; CI: 20.3 - 24.6%) (Figure 1, Table S3).

We analysed prescribing prevalence for any of the five antiplatelets: aspirin, clopidogrel, dipyridamole, prasugrel and ticagrelor. Like the prescribing trend of anticoagulants, patients with liver disease had a lower rate of antiplatelet prescribing compared with those without liver disease (56.2% [2,207/3,927] vs. 71.1% [249,258/350,803]). In individuals with liver disease, the highest prevalence was observed in autoimmune liver disease (61.5% [195/317]; CI: 56.2 - 66.9%) and the lowest was in HCV (38.8% [145/374]; CI: 33.8 - 43.7%) (Figure 1, Table S3). For other liver conditions, prescribing prevalence for antiplatelets were as follow: ALD (54.9% [899/1,639]; CI: 52.4 - 57.3%), cirrhosis (55.7% [886/1,592]; CI: 53.2 - 58.1%), NAFLD (56.3% [802/1,424]; CI: 53.7 - 58.9%) and HBV (57.3% [145/253]; CI: 51.2 - 63.4%). Regional variations in prescribing prevalence for anticoagulants and antiplatelets were investigated and reported in the supplementary appendix.

### 3.2 Baseline characteristics of individuals with at least one prescription

Individuals with at least one prescription were included in adherence and persistence analyses. For anticoagulants, this involved 806 individuals with liver disease and 103,222 without liver disease. For antiplatelets, 2,207 individuals with liver disease and 249,258 individuals without liver disease were included in the analyses. Baseline characteristics of individuals with at least one prescription were investigated (Table S1 and Table S2). The average age of individuals at the time of first anticoagulant prescription was 70.8 years and 74.6 years in patients with and without liver disease, respectively. Among all individuals with liver disease who had at least one anticoagulant prescription, 62.0% [500/806] were men and 38% [306/806] were women (Table S1). Among all individuals without liver disease who had at least one anticoagulant prescription, 55.9% [57709/103222] were men and 44.1% [45513/103222] were women (Table S2). Individuals with higher CHA_2_DS_2_VASc scores (score 3 and above) were more likely to be prescribed anticoagulants in both groups. Like the results on anticoagulant prescribing, patients with liver disease encountered their first antiplatelet prescription at a younger age (65.7 years) compared with those without liver disease (70.9 years) (Table S1 and Table S2).

### 3.3 Patients with liver disease, when prescribed antithrombotic medications, had higher adherence to these drugs compared with individuals without liver disease

**Figure 2:**
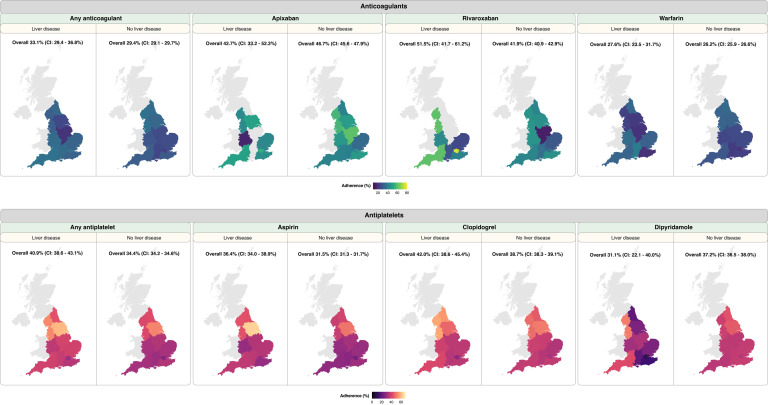
Adherence to antithrombotic medications in individuals with or without liver disease. Adherence was estimated by the proportion of days covered (PDC) over 12 months following the first prescription. Patients having PDC > 80% were considered adherent and maps depict the percentage of patients who were adherent in each geographical region. Overall adherence for England is annotated above each map. CI: 95% confidence interval.

Although patients with liver disease had a lower prescribing prevalence, patients who ended up being prescribed antithrombotic medications and had at least 12 months of follow-up had higher adherence compared with people without liver disease: anticoagulants (33.1% [208/628] vs. 29.4% [26,615/90,569]) and antiplatelets (40.9% [743/1,818] vs. 34.4% [76,834/223,154]) (Figure 2, Table S4). For specific anticoagulants, adherence to rivaroxaban and warfarin were also found to be higher in patients with liver disease: rivaroxaban (51.5% [52/101] vs. 41.9% [3,828/9,135]) and warfarin (27.6% [125/453] vs. 26.2% [20,302/77,370]). For apixaban, however, adherence was higher in people without liver disease (46.7% [3,544/7,584]) compared with those with liver disease (42.7% [44/103]) (Figure 2, Table S4). When analysing adherence for specific antiplatelets, we observed that patients with liver disease had a higher rate of adherence to aspirin (36.4% [540/1,482] vs. 31.5% [62,276/197,656]) and clopidogrel (42.0% [340/810] vs. 38.7% [27,870/72,016]) compared with those without liver disease. For dipyridamole, however, the opposite pattern was observed, individuals without liver disease had higher adherence (37.2% [6,585/17,681] in people without liver disease vs. 31.1% [32/103] in people with liver disease) (Figure 2, Table S4). Geographical variations in adherence were investigated and reported in the supplementary appendix.

### 3.4 Likelihood of non-adherence

In patients with liver disease, multivariable analysis revealed that the likelihoods of non-adherence to apixaban and rivaroxaban were lower than warfarin at both 6 and 12 months. Relative to warfarin, the likelihoods of non-adherence were as follow: apixaban (6 months odds ratio (OR) 0.52, CI: 0.34-0.78, p=0.0015; 12 months OR 0.51, CI: 0.33-0.80, p=0.0029) and rivaroxaban (6 months OR 0.44, CI: 0.29-0.67, p<0.0001; 12 months OR 0.36, CI: 0.23-0.56, p<0.0001) (Table 1, Table S6). Female gender was associated with a reduced likelihood of non-adherence at 6 months (OR 0.61, CI: 0.44-0.83, p=0.0018) and 12 months (OR 0.64, CI: 0.46-0.91, p=0.011). Increasing comorbidity burden (by CHA_2_DS_2_VASc score) was associated with a decreased likelihood of non-adherence especially at 12 months: CHA_2_DS_2_VASc scores 3-4 (OR 0.53, CI:0.30-0.91, p=0.024) and scores 5-9 (OR 0.44, CI: 0.24-0.77, p=0.0052) compared with scores 0-1. Chronic kidney disease was associated with a decreased likelihood of non-adherence at 12 months (OR: 0.65, CI: 0.45-0.95, p=0.025). However, the presence of cirrhosis and liver-related complications (i.e., ascites, hepatic encephalopathy and varices) were not associated with non-adherence to anticoagulants (Table 1). Patients with TTR > 60% had a lower risk of non-adherence (OR 0.52, CI: 0.31-0.87, p=0.013) to warfarin at 12 months.

**Table 1:** Likelihood of non-adherence to antithrombotic therapy at 6 months and 12 months in patients with liver disease. Adjusted odds ratios (ORs) are reported.

	Anticoagulants (6 months)				Anticoagulants (12 months)					Antiplatelets (6 months)				Antiplatelets (12 months)			
	OR	Lower CI	Upper CI	P value	OR	Lower CI	Upper CI	P value		OR	Lower CI	Upper CI	P value	OR	Lower CI	Upper CI	P value
**Female**	0.61	0.44	0.83	**0.0018**	0.64	0.46	0.91	**0.011**	**Female**	0.79	0.66	0.95	**0.014**	0.84	0.69	1.02	0.072
**Age**									**Age**								
Age 30 - 49	1.00 (ref)				1.00 (ref)				Age 30 - 49	1.00 (ref)				1.00 (ref)			
Age 50 - 59	2.71	1.05	7.06	**0.038**	1.28	0.41	3.67	0.66	Age 50 - 59	0.92	0.65	1.30	0.64	0.74	0.51	1.07	0.11
Age 60 - 69	1.62	0.68	3.82	0.27	0.85	0.29	2.20	0.76	Age 60 - 69	0.88	0.63	1.24	0.48	0.77	0.54	1.09	0.15
Age 70 - 79	1.42	0.61	3.31	0.41	0.80	0.28	2.03	0.65	Age 70 - 79	0.90	0.64	1.28	0.57	0.78	0.54	1.11	0.17
Age 80 & above	0.93	0.39	2.23	0.87	0.42	0.14	1.09	0.09	Age 80 & above	0.48	0.32	0.71	**0.00033**	0.49	0.32	0.75	**0.0011**
**Comorbidities**									**Comorbidities**								
Chronic kidney disease	0.77	0.55	1.09	0.14	0.65	0.45	0.95	**0.025**	Chronic kidney disease	0.72	0.56	0.91	**0.0054**	0.77	0.60	0.98	**0.037**
Ascites	1.16	0.75	1.82	0.51	1.12	0.69	1.84	0.66	Ascites	1.03	0.81	1.32	0.79	1.14	0.88	1.48	0.34
Cirrhosis	1.25	0.92	1.71	0.15	1.23	0.88	1.73	0.23	Cirrhosis	1.17	0.98	1.40	0.086	1.24	1.02	1.50	**0.027**
Hepatic encephalopathy	0.90	0.47	1.75	0.75	1.09	0.53	2.35	0.83	Hepatic encephalopathy	0.80	0.57	1.13	0.20	0.96	0.67	1.39	0.84
Varices	1.49	0.86	2.64	0.16	1.70	0.94	3.29	0.093	Varices	1.25	0.94	1.66	0.12	1.34	1.00	1.82	0.056
Proton pump inhibitor use	0.82	0.60	1.12	0.21	0.99	0.71	1.38	0.94	Proton pump inhibitor use	0.73	0.61	0.88	**0.0010**	0.79	0.65	0.96	**0.017**
**CHA_2_DS_2_ VASc score**									**CHA_2_DS_2_ VASc score**								
0-1	1.00 (ref)				1.00 (ref)				0-1	NA				NA			
2	0.70	0.39	1.23	0.22	0.63	0.33	1.19	0.16	2	NA				NA			
3-4	0.69	0.43	1.12	0.14	0.53	0.30	0.91	**0.024**	3-4	NA				NA			
5-9	0.51	0.30	0.83	**0.0080**	0.44	0.24	0.77	**0.0052**	5-9	NA				NA			
**Child-Pugh score**									**Child-Pugh score**								
Class A (score 5-6)	1.00 (ref)				1.00 (ref)				Class A (score 5-6)	1.00 (ref)				1.00 (ref)			
Class B (score 7-9)	1.23	0.89	1.69	0.21	1.10	0.78	1.55	0.60	Class B (score 7-9)	1.10	0.91	1.34	0.32	1.11	0.91	1.36	0.30
Class C (score 10-15)	0.75	0.14	4.11	0.73	1.19	0.86	2.85	0.97	Class C (score 10-15)	1.36	0.64	3.01	0.43	1.15	0.52	2.63	0.74
**FIB-4 score**									**FIB-4 score**								
< 1.45	1.00 (ref)				1.00 (ref)				< 1.45	1.00 (ref)				1.00 (ref)			
1.45-3.25	0.83	0.60	1.14	0.25	0.87	0.61	1.23	0.43	1.45-3.25	0.84	0.69	1.01	0.066	0.88	0.72	1.07	0.20
>3.25	0.59	0.33	1.04	0.066	0.68	0.37	1.26	0.21	>3.25	1.18	0.83	1.69	0.35	1.48	1.00	2.22	0.056
**Warfarin time in therapeutic range (TTR)**																	
TTR > 60%	0.73	0.46	1.15	0.18	0.52	0.31	0.87	**0.013**									
**Medication type**									**Medication type**								
Warfarin	1.00 (ref)				1.00 (ref)				Aspirin	1.00 (ref)				1.00 (ref)			
Apixaban	0.52	0.34	0.78	**0.0015**	0.51	0.33	0.80	**0.0029**	Clopidogrel	0.72	0.61	0.85	**0.00011**	0.79	0.67	0.94	**0.0092**
Rivaroxaban	0.44	0.29	0.67	**< 0.0001**	0.36	0.23	0.56	**< 0.0001**	Dipyridamole	0.92	0.63	1.37	0.68	1.27	0.83	1.98	0.27

For antiplatelets, the likelihood of non-adherence with clopidogrel was lower than with aspirin at both 6 months (OR 0.72, CI: 0.61-0.85, p=0.00011) and 12 months (OR 0.79, CI: 0.67-0.94, p=0.0092) (Table 1). Females had a lower likelihood of non-adherence with antiplatelets at 6 months (OR 0.79, CI: 0.66-0.95, p=0.014). Individuals aged 80 and above were less likely to be non-adherent compared with younger individuals at 6 months (OR 0.48, CI: 0.32-0.71, p=0.00033) and 12 months (OR 0.49, CI: 0.32-0.75, p=0.0011). Chronic kidney disease was associated with decreased risk of non-adherence with antiplatelets (6 months OR 0.72, CI: 0.56-0.91, p=0.0054; 12 months OR 0.77, CI: 0.60-0.98, p=0.037). In contrast, cirrhosis was associated with an increased likelihood of non-adherence with antiplatelets at 12 months (OR 1.24, CI: 1.02-1.50, p=0.027). Adherence to antithrombotic therapy does not appear to be affected by liver disease severity as measured by Child-Pugh and FIB-4 scores (Table 1). Proton-pump inhibitor use was associated with lower risk of non-adherence with antiplatelets at 6 months (OR 0.73, CI: 0.61-0.88, p=0.0010) and 12 months (OR 0.79, CI: 0.65-0.96, p=0.017) (Table 1).

### 3.5 Persistence with antithrombotic medications was similar between patients with and without liver disease

**Figure 3:**
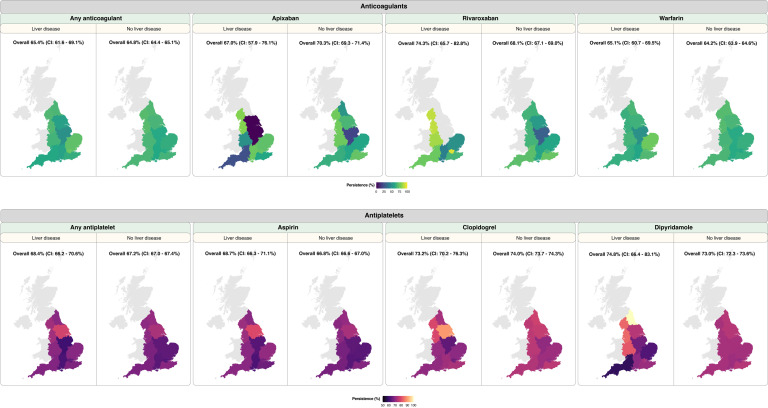
Persistence with antithrombotic medications in individuals with or without liver disease. Individuals were considered persistent until a prescription gap of > 90 days. Maps depict the percentage of patients who were persistent at 12 months following the first prescription in each geographical region. Overall persistence for England is annotated above each map. CI: 95% confidence interval.

Overall, persistence at 12 months for any anticoagulants was similar at 65.4% [402/615] and 64.8% [57,642/89,022] in patients with and without liver disease, respectively (Figure 3, Table S5). For antiplatelets, persistence was 68.4% [1,175/1,718] and 67.2% [142,855/212,448] in patients with and without liver disease, respectively. When considering specific anticoagulant medications, patients with liver disease had a higher persistence with rivaroxaban (74.3% [75/101] vs. 68.1% [6,217/9,135]) and warfarin (65.1% [295/453] vs. 64.2% [49,687/77,370]) compared with those without liver disease. For apixaban, persistence was 67.0% [69/103] and 70.3% [5,334/7,584] in patients with and without liver disease, respectively. Persistence analyses on specific antiplatelets in patients with or without liver disease were as follow: aspirin (68.7% [1,018/1,482] vs. 66.8% [131,953/197,656]), clopidogrel (73.2% [593/810] vs. 74.0% [53,298/72,016]) and dipyridamole (74.8% [77/103] vs. 73.0% [12,904/17,681]) (Figure 3, Table S5). Geographical variations in persistence were investigated and reported in the supplementary appendix.

### 3.6 Risk of non-persistence

Multivariable analyses in patients with liver disease undergoing anticoagulant therapy demonstrated that rivaroxaban had a lower risk of non-persistence at 12 months (hazard ratio (HR) 0.64, CI: 0.42-0.97, p=0.035), relative to warfarin (Table 2, Table S6). Females experienced a lower risk of non-persistence at 12 months (HR 0.74, CI: 0.56-0.98, p=0.038) compared with males. Patients with higher CHA_2_DS_2_VASc scores had a decreased risk of non-persistence compared with those with scores 0-1. At 6 months, the risk of non-persistence for patients with scores 3-4 was 0.57 (CI: 0.37-0.87, p=0.010) and for patients with scores 5-9, HR was 0.55 (CI: 0.34-0.88, p=0.012). Similarly at 12 months, risks of non-persistence were as follow: scores 3-4 (HR 0.56, CI: 0.39-0.80, p=0.0015) and scores 5-9 (HR 0.64, CI: 0.44-0.94, p=0.023) (Table 2). Patients with varices also had an increased risk of non-persistence (HR: 1.50, CI: 1.02-2.25, p=0.047). For antiplatelets, patients treated with clopidogrel had a lower risk of non-persistence at 6 months (HR 0.72, CI: 0.58-0.89, p=0.0025) and 12 months (HR 0.81, CI: 0.69-0.95, p=0.010), relative to aspirin (Table 2). Sex, age and renal and liver-related comorbidities were not associated with non-persistence with antiplatelets (Table 2). Patients with Child-Pugh Class B (relative to Class A) had a higher risk of non-persistence with antiplatelet therapy at both 6 months (HR 1.41, CI: 1.14-1.73, p=0.0015) and 12 months (HR 1.27, CI:1.07-1.51, p=0.0055) (Table 2). Patients with TTR > 60% had a lower risk of non-persistence (HR 0.64, CI: 0.42-0.98, p=0.039) to warfarin at 12 months. Proton-pump inhibitor use was also associated with lower risk of non-persistence with antiplatelets at 6 months (HR 0.79, CI: 0.64-0.97, p=0.024) and 12 months (HR 0.80, CI: 0.68-0.94, p=0.0065) (Table 2).

**Table 2:** Risk of non-persistence to antithrombotic therapy at 6 months and 12 months in patients with liver disease. Adjusted hazard ratios (HRs) are reported.

	Anticoagulants (6 months)				Anticoagulants (12 months)					Antiplatelets (6 months)				Antiplatelets (12 months)			
	HR	Lower CI	Upper CI	P value	HR	Lower CI	Upper CI	P value		HR	Lower CI	Upper CI	P value	HR	Lower CI	Upper CI	P value
**Female**	0.72	0.51	1.02	0.061	0.74	0.56	0.98	**0.038**	**Female**	1.00	0.80	1.23	0.96	1.00	0.85	1.19	0.97
**Age**									**Age**								
Age 30 - 49	1.00 (ref)				1.00 (ref)				Age 30 - 49	1.00 (ref)				1.00 (ref)			
Age 50 - 59	1.16	0.43	3.13	0.77	1.05	0.48	2.31	0.91	Age 50 - 59	0.84	0.58	1.21	0.35	0.78	0.58	1.06	0.11
Age 60 - 69	1.07	0.42	2.71	0.89	0.97	0.46	2.02	0.93	Age 60 - 69	0.80	0.56	1.15	0.23	0.85	0.64	1.13	0.26
Age 70 - 79	1.29	0.52	3.21	0.58	1.05	0.51	2.16	0.90	Age 70 - 79	0.73	0.50	1.06	0.10	0.82	0.61	1.11	0.20
Age 80 & above	0.93	0.35	2.42	0.87	0.92	0.43	1.95	0.82	Age 80 & above	0.79	0.51	1.22	0.29	0.84	0.59	1.19	0.33
**Comorbidities**									**Comorbidities**								
Chronic kidney disease	1.01	0.71	1.45	0.94	0.90	0.67	1.22	0.51	Chronic kidney disease	0.79	0.59	1.05	0.10	0.87	0.70	1.09	0.23
Ascites	1.36	0.90	2.07	0.14	1.10	0.76	1.59	0.63	Ascites	1.11	0.85	1.45	0.45	1.13	0.91	1.40	0.26
Cirrhosis	1.16	0.84	1.60	0.36	1.22	0.94	1.59	0.13	Cirrhosis	1.24	1.01	1.52	0.04	1.09	0.92	1.28	0.32
Hepatic encephalopathy	0.79	0.37	1.68	0.54	0.92	0.51	1.64	0.77	Hepatic encephalopathy	0.91	0.61	1.36	0.64	1.05	0.77	1.42	0.77
Varices	1.44	0.88	2.36	0.15	1.50	1.01	2.25	**0.047**	Varices	1.05	0.77	1.44	0.74	1.19	0.94	1.51	0.16
Proton pump inhibitor use	0.85	0.62	1.17	0.31	1.01	0.77	1.31	0.95	Proton pump inhibitor use	0.79	0.64	0.97	**0.024**	0.80	0.68	0.94	**0.0065**
**CHA_2_DS_2_ VASc score**									**CHA_2_DS_2_ VASc score**								
0-1	1.00 (ref)				1.00 (ref)				0-1	NA				NA			
2	0.78	0.47	1.29	0.33	0.66	0.43	1.02	0.064	2	NA				NA			
3-4	0.57	0.37	0.87	**0.010**	0.56	0.39	0.80	**0.0015**	3-4	NA				NA			
5-9	0.55	0.34	0.88	**0.012**	0.64	0.44	0.94	**0.023**	5-9	NA				NA			
**Child-Pugh score**									**Child-Pugh score**								
Class A (score 5-6)	1.00 (ref)				1.00 (ref)				Class A (score 5-6)	1.00 (ref)				1.00 (ref)			
Class B (score 7-9)	0.77	0.55	1.09	0.14	0.76	0.58	1.01	0.058	Class B (score 7-9)	1.41	1.14	1.73	**0.0015**	1.27	1.07	1.51	**0.0055**
Class C (score 10-15)	0.60	0.08	4.27	0.61	0.41	0.06	2.90	0.37	Class C (score 10-15)	0.91	0.38	2.21	0.84	0.94	0.46	1.88	0.85
**FIB-4 score**									**FIB-4 score**								
< 1.45	1.00 (ref)				1.00 (ref)				< 1.45	1.00 (ref)				1.00 (ref)			
1.45-3.25	0.93	0.67	1.30	0.68	1.10	0.83	1.45	0.51	1.45-3.25	1.06	0.85	1.32	0.60	1.14	0.96	1.35	0.15
>3.25	0.85	0.46	1.57	0.60	1.10	0.68	1.78	0.71	>3.25	1.30	0.91	1.86	0.15	1.27	0.94	1.71	0.12
**Warfarin time in therapeutic range (TTR)**																	
TTR > 60%	0.65	0.38	1.11	0.11	0.64	0.42	0.98	**0.039**									
**Medication type**									**Medication type**								
Warfarin	1.00 (ref)				1.00 (ref)				Aspirin	1.00 (ref)				1.00 (ref)			
Apixaban	0.88	0.57	1.38	0.58	0.84	0.58	1.22	0.37	Clopidogrel	0.72	0.58	0.89	**0.0025**	0.81	0.69	0.95	**0.010**
Rivaroxaban	0.77	0.48	1.23	0.27	0.64	0.42	0.97	**0.035**	Dipyridamole	0.83	0.51	1.35	0.44	0.77	0.52	1.15	0.20

### 3.7 Adherence and persistence

Primary non-adherence (stopping after the first prescription) to anticoagulants was higher in people with liver disease (7.9% [64/806]) compared with individuals without liver disease (4.7% [4,841/103,222]). Primary non-adherence to antiplatelets was also higher in people with liver disease (6.2% [137/2,207]) compared with those free of liver disease (4.4% [10,993/249,258]).

Among individuals with ≥ 6 months of data, patients with liver disease, compared with individuals without liver disease, were more adherent to and persistent with rivaroxaban (54.8% [63/115] vs. 48.9% [4,721/9,662]) and warfarin (34.9% [168/482] vs. 33.6% [27,017/80,390]) but not with apixaban (46.6% vs. 54.4%). Non-adherence, non-persistence was the highest with warfarin and lowest with apixaban: patients with liver disease (warfarin 21.0% [101/482], apixaban 15.3% [18/118]) and patients without liver disease (warfarin 21.5% [17,288/80,390], apixaban 12.5% [1,033/8,241]) (Table 3). Among individuals with ≥ 12 months of data, a similar trend of patients with liver disease being more adherent to and persistent with rivaroxaban and warfarin was observed, compared with patients without liver disease. Non-adherence, non-persistence was also the highest with warfarin: patients with liver disease (34.2% [155/453]) and patients without liver disease (35.0% [27,074/77,370]) (Table 3).

**Table 3:** Adherence and persistence to antithrombotic therapy at 6 months and 12 months in patients with or without chronic liver disease (CLD).

	Anticoagulants					
	Apixaban		Rivaroxaban		Warfarin	
	With CLD	Without CLD	With CLD	Without CLD	With CLD	Without CLD
	**Adherence and persistence at 6 months**					
**Patients with at least 6 months of follow-up**	118	8241	115	9662	482	80390
**Adherent (%)**	61 (51.7)	4842 (58.8)	64 (55.7)	5095 (52.7)	172 (35.7)	27803 (34.6)
**Persistent (%)**	94 (79.7)	6846 (83.1)	94 (81.7)	7905 (81.8)	377 (78.2)	62316 (77.5)
**Adherent, persistent (%)**	55 (46.6)	4480 (54.4)	63 (54.8)	4721 (48.9)	168 (34.9)	27017 (33.6)
**Adherent, non-persistent (%)**	6 (5.1)	362 (4.4)	1 (0.9)	374 (3.9)	4 (0.8)	786 (1.0)
**Non-adherent, persistent (%)**	39 (33.1)	2366 (28.7)	31 (27.0)	3184 (33.0)	209 (43.4)	35299 (43.9)
**Non-adherent, non-persistent (%)**	18 (15.3)	1033 (12.5)	20 (17.4)	1383 (14.3)	101 (21.0)	17288 (21.5)
	**Adherence and persistence at 12 months**					
**Patients with at least 12 months of follow-up**	103	7584	101	9135	453	77370
**Adherent (%)**	44 (42.7)	3544 (46.7)	52 (51.5)	3828 (41.9)	125 (27.6)	20302 (26.2)
**Persistent (%)**	69 (67.0)	5334 (70.3)	75 (74.3)	6217 (68.1)	295 (65.1)	49687 (64.2)
**Adherent, persistent (%)**	39 (37.9)	3147 (41.5)	50 (49.5)	3351 (36.7)	122 (26.9)	19693 (25.5)
**Adherent, non-persistent (%)**	5 (4.9)	397 (5.2)	2 (2.0)	477 (5.2)	3 (0.7)	609 (0.8)
**Non-adherent, persistent (%)**	30 (29.1)	2187 (28.8)	25 (24.8)	2866 (31.4)	173 (38.2)	29994 (38.8)
**Non-adherent, non-persistent (%)**	29 (28.2)	1853 (24.4)	24 (23.8)	2441 (26.7)	155 (34.2)	27074 (35.0)

	Antiplatelets					
	Aspirin		Clopidogrel		Dipyridamole	
	With CLD	Without CLD	With CLD	Without CLD	With CLD	Without CLD

	**Adherence and persistence at 6 months**					
**Patients with at least 6 months of follow-up**	1582	204369	871	74338	111	18115
**Adherent (%)**	653 (41.3)	70920 (34.7)	430 (49.4)	34768 (46.8)	48 (43.2)	7995 (44.1)
**Persistent (%)**	1290 (81.5)	156873 (76.8)	751 (86.2)	62182 (83.6)	95 (85.6)	14828 (81.9)
**Adherent, persistent (%)**	639 (40.4)	69812 (34.2)	414 (47.5)	31779 (42.7)	47 (42.3)	7219 (39.9)
**Adherent, non-persistent (%)**	14 (0.9)	1108 (0.5)	16 (1.8)	2989 (4.0)	1 (0.9)	776 (4.3)
**Non-adherent, persistent (%)**	651 (41.2)	87061 (42.6)	337 (38.7)	30403 (40.9)	48 (43.2)	7609 (42.0)
**Non-adherent, non-persistent (%)**	278 (17.6)	46388 (22.7)	104 (11.9)	9167 (12.3)	15 (13.5)	2511 (13.9)
	**Adherence and persistence at 12 months**					
**Patients with at least 12 months of follow-up**	1482	197656	810	72016	103	17681
**Adherent (%)**	540 (36.4)	62276 (31.5)	340 (42.0)	27870 (38.7)	32 (31.1)	6585 (37.2)
**Persistent (%)**	1018 (68.7)	131953 (66.8)	593 (73.2)	53298 (74.0)	77 (74.8)	12904 (73.0)
**Adherent, persistent (%)**	526 (35.5)	60909 (30.8)	315 (38.9)	24547 (34.1)	28 (27.2)	5753 (32.5)
**Adherent, non-persistent (%)**	14 (0.9)	1367 (0.7)	25 (3.1)	3323 (4.6)	4 (3.9)	832 (4.7)
**Non-adherent, persistent (%)**	492 (33.2)	71044 (35.9)	278 (34.3)	28751 (39.9)	49 (47.6)	7151 (40.4)
**Non-adherent, non-persistent (%)**	450 (30.4)	64336 (32.5)	192 (23.7)	15395 (21.4)	22 (21.4)	3945 (22.3)

For antiplatelet medications, among patients with ≥ 6 months of data, patients with liver disease compared with those without liver disease were more adherent to and persistent with aspirin (40.4% [639/1,582] vs. 34.2% [69,812/204,369]), clopidogrel (47.5% [414/871] vs. 42.7% [31,779/74,338]) and dipyridamole (42.3% [47/111] vs. 39.9% [7,219/18,115]). Non-adherence, non-persistence was the highest with aspirin (with liver disease: 17.6% [278/1,582]; without liver disease: 22.7% [46,388/204,369]) and the lowest with clopidogrel (with liver disease: 11.9% [104/871]; without liver disease: 12.3% [9,167/74,338]) (Table 3). Among patients with ≥ 12 months of data, patients with liver disease had higher adherence and persistence with aspirin (35.5% [526/1,482] vs. 30.8% [60,909/197,656]) and clopidogrel (38.9% [315/810] vs., 34.1% [24,547/72,016]), but not with dipyridamole (27.2% [28/103] vs. 32.5% [5,753/17,681]) which was higher in patients without liver disease. Non-adherence, non-persistence was again the highest with aspirin (with liver disease: 30.4% [450/1,482]; without liver disease: 32.5% [64,336/197,656]]) compared with clopidogrel or dipyridamole (Table 3).

### 3.8 Effect of adherence on the risk of stroke and bleeding

We explored the impact of adherence to antithrombotic therapy on the risk of stroke (efficacy) and bleeding (safety). In patients without liver disease, not taking anticoagulants for 3 to 6 months (HR 1.22, CI: 1.16-1.27, p<0.0001) and > 6 months (HR 1.20, CI: 1.15-1.25, p<0.0001) were associated with an elevated risk of stroke (Table 4, Table S7). Observations on increased stroke risk were replicated when stratifying by CHA_2_DS_2_VASc score where patients not taking anticoagulants for ≥ 3 months had higher risk regardless of their score, compared with those not taking anticoagulants for < 1 week. HRs in patients not taking anticoagulants for > 6 months were: CHA_2_DS_2_VASc scores 0-1 (1.37, CI: 1.15-1.62, p<0.0001), score 2 (1.37, CI: 1.20-1.56, p<0.0001), scores 3-4 (1.27, CI: 1.19-1.35, p<0.0001) and scores 5-9 (1.18, CI: 1.12-1.26, p<0.0001). In patients without liver disease, an increase in adherence was associated with an increased risk of non-fatal bleeding (HR 1.08 per 10% increase in PDC, CI: 1.02-1.14, p=0.012). When investigating the impact of adherence on stroke risk in patients on antiplatelet therapy, we observed similar results on non-adherence and increased risk in patients without liver disease. Individuals not taking antiplatelets for 3 to 6 months (HR 1.11, CI: 1.09-1.14, p<0.0001) and > 6 months (HR 1.32, CI: 1.29-1.34, p<0.0001) had a higher risk of stroke compared with people not taking antiplatelets for < 1 week. Adherence to antiplatelets in patients without liver disease was, however, associated with an increased risk of bleeding (HR 1.18, CI: 1.14-1.22, p<0.0001). A separate analysis on patients with liver disease was not performed because of the lack of an adequate number of events in this population to provide sufficient power for a meaningful analysis in these patients.

**Table 4:** Impact of adherence to antithrombotic therapy on risk of stroke (efficacy) and bleeding (safety) in patients without chronic liver disease. Adjusted hazard ratios (HRs) are reported.

	A) Anticoagulant therapy				
	Outcome = Ischaemic stroke				
	HR	Lower CI	Upper CI		P value
**Time not taking medication**					
**All patients**					
< 1 week	1.00 (ref)				
1 week to 1 month	0.993	0.919	1.072		0.85
1 to 3 months	1.049	0.958	1.149		0.31
3 to 6 months	1.216	1.164	1.270		**< 0.0001**
> 6 months	1.200	1.152	1.249		**< 0.0001**
**CHA_2_DS_2_ VASc score 0-1**					
< 1 week	1.00 (ref)				
1 week to 1 month	1.272	0.944	1.715		0.11
1 to 3 months	1.246	0.858	1.810		0.25
3 to 6 months	1.349	1.123	1.619		**0.0013**
> 6 months	1.367	1.152	1.622		**< 0.0001**
**CHA_2_DS_2_ VASc score 2**					
< 1 week	1.00 (ref)				
1 week to 1 month	0.998	0.785	1.269		0.99
1 to 3 months	1.091	0.810	1.469		0.57
3 to 6 months	1.435	1.254	1.642		**< 0.0001**
> 6 months	1.367	1.202	1.555		**< 0.0001**
**CHA_2_DS_2_ VASc score 3-4**					
< 1 week	1.00 (ref)				
1 week to 1 month	0.976	0.863	1.105		0.70
1 to 3 months	1.084	0.937	1.253		0.28
3 to 6 months	1.195	1.112	1.283		**< 0.0001**
> 6 months	1.266	1.185	1.354		**< 0.0001**
**CHA_2_DS_2_ VASc score 5-9**					
< 1 week	1.00 (ref)				
1 week to 1 month	1.011	0.900	1.137		0.85
1 to 3 months	1.020	0.891	1.168		0.77
3 to 6 months	1.154	1.082	1.231		**< 0.0001**
> 6 months	1.183	1.115	1.255		**< 0.0001**


	Outcome = Non-fatal bleeding				
	HR	Lower CI	Upper CI	P value	
	**Without CLD**				
**Per 10% increase in adherence (PDC)**	1.079	1.017	1.144	**0.012**	

	B) Antiplatelet therapy				
	Outcome = Ischaemic stroke				
	HR	Lower CI	Upper CI		P value
**Time not taking medication**					
< 1 week	1.00 (ref)				
1 week to 1 month	0.925	0.888	1.064		0.067
1 to 3 months	1.046	0.994	1.101		0.086
3 to 6 months	1.111	1.086	1.136		**< 0.0001**
> 6 months	1.315	1.287	1.343		**< 0.0001**

	Outcome = Non-fatal bleeding				
	HR	Lower CI	Upper CI	P value	
**Per 10% increase in adherence (PDC)**	1.183	1.144	1.224	**< 0.0001**	

In order to assess the impact of adherence in patients with liver disease, we performed additional analyses to assess stroke outcomes comparing all patients with liver disease versus patients without liver disease (as the reference). For analyses on stroke risks, we stratified patients (with and without liver disease) according to the time patients spent not taking their medication. We observed that patients with liver disease, compared with those without liver disease do not appear to experience any increase in stroke risk when considering anticoagulant therapy (Table 5, Table S8). However, when considering antiplatelet therapy, patients with liver disease who spent < 1 week not taking their antiplatelet medication had a higher risk of stroke compared with patients without liver disease (HR 1.45, CI: 1.19-1.78, p=0.00030). Similarly, patients with liver disease compared with those without liver disease, experienced a higher risk of stroke when they stopped taking their antiplatelet medication for 3 to 6 months (HR 1.42, CI: 1.14-1.77, p=0.0017) and for more than 6 months (HR 1.30, CI: 1.12-1.52, p=0.00082) (Table 5). We next analysed bleeding risks among patients who were adherent (PDC > 80%). Adherence to anticoagulants was not associated with an increased bleeding risk in patients with liver disease compared with those without liver disease (Table 5). In contrast, adherence to antiplatelets was associated with an increased bleeding risk in patients with liver disease (HR 2.02, CI: 1.73-2.36, p<0.0001) (Table 5).

**Table 5:** Impact of adherence to antithrombotic therapy on risk of stroke and bleeding in patients with chronic liver disease (CLD) compared with those without CLD as a reference. Analyses for risk of stroke were performed based on patients stratified according to the time they spent not taking medications. Analyses for risk of bleeding were performed in patients who were adherent. Adjusted hazard ratios (HRs) are reported.

Anticoagulant therapy					
	HR	Lower CI	Upper CI	P value	Outcome
	**Not taking medication for < 1 week**				
With CLD	1.435	0.943	2.182	0.092	Stroke
	**Not taking medication for 1 week to 1 month**				
With CLD	0.802	0.258	2.498	0.70	Stroke
	**Not taking medication for 1 month to 3 months**				
With CLD	1.129	0.281	4.534	0.86	Stroke
	**Not taking medication for 3 months to 6 months**				
With CLD	1.097	0.649	1.854	0.73	Stroke
	**Not taking medication for > 6 months**				
With CLD	1.143	0.852	1.533	0.37	Stroke
	**Patients who were adherent (PDC > 80%)**				
With CLD	1.338	0.959	1.866	0.086	Bleeding

Antiplatelet therapy					
	HR	Lower CI	Upper CI	P value	Outcome

	**Not taking medication for < 1 week**				
With CLD	1.454	1.187	1.781	**0.00030**	Stroke
	**Not taking medication for 1 week to 1 month**				
With CLD	1.587	0.954	2.638	0.075	Stroke
	**Not taking medication for 1 month to 3 months**				
With CLD	1.058	0.549	2.038	0.87	Stroke
	**Not taking medication for 3 months to 6 months**				
With CLD	1.422	1.141	1.772	**0.0017**	Stroke
	**Not taking medication for > 6 months**				
With CLD	1.303	1.116	1.521	**0.00082**	Stroke
	**Patients who were adherent (PDC > 80%)**				
With CLD	2.021	1.729	2.363	**< 0.0001**	Bleeding

## 4 Discussion

This study is the first to investigate antithrombotic prescribing prevalence, adherence, persistence, safety and efficacy in patients with and without liver disease using a single nationwide cohort. First, we observed an overall trend of lower prescribing prevalence of anticoagulants and antiplatelets in patients with liver disease compared with those without liver disease. Prescribing prevalence was on average 38% and 21% lower for anticoagulants and antiplatelets respectively in patients with liver disease, with heterogeneity across liver disease types. Second, our study demonstrates that adherence to any anticoagulation or antiplatelet therapy was suboptimal in both patients with and without liver disease. Less than 50% of patients adhered to their medications at 1 year. Primary non-adherence to antithrombotic therapy was more common in patients with liver disease. Third, patients with liver disease were more likely to adhere to direct oral anticoagulants (DOACs), i.e., apixaban and rivaroxaban, than to warfarin. There was an increased likelihood of adherence to clopidogrel, but not dipyridamole, compared with aspirin. Fourth, higher CHA_2_DS_2_VASc scores were associated with reduced risk of non-adherence and non-persistence with anticoagulants. Fifth, better adherence to anticoagulants and antiplatelets was associated with lower stroke risk and a small increase in bleeding risk in patients without liver disease. Sixth, poor adherence to antiplatelets was associated with higher stroke risk in patients with liver disease compared with those without liver disease. Adherence to antiplatelets in patients with liver disease was, however, linked to increase in bleeding risk.

### 4.1 Management of antithrombotic therapy in patients with liver disease

Patients with liver disease are excluded from major randomised trials on antithrombotic medicines as they are often contraindicated and are at a higher risk of bleeding. The scarcity of evidence from trials is further exacerbated by limited real-world evidence on adherence to these drugs. Non-adherence has been a major issue with long-term pharmacological therapy and adherence is even harder to achieve in patients with contraindications. Furthermore, common barriers to antithrombotic medication prescribing include clinicians not being fully familiar with the bleeding and thrombotic homeostasis in patients with liver disease.

Guidelines from NHS trusts [30,31] stated that warfarin is the preferred choice of treatment in patients with elevated liver enzymes and hepatic impairment due to the lack of data from DOAC clinical trials. But in general, any antithrombotic should be used with caution if coagulopathy and thrombocytopenia are evident. We found that adherence to rivaroxaban was higher in patients with liver disease than those without liver disease, and adherence to apixaban and rivaroxaban was higher than warfarin. Another study demonstrated that in patients with prior liver disease and chronic alcoholism, rivaroxaban and apixaban use, relative to warfarin, was associated with a lower risk of hospitalisation for acute liver injury [32]. A meta-analyses on clinical trials found no increase in the risk of drug-induced liver injury when comparing DOACs with warfarin [33]. Similarly, a report on Canadian patients found no difference in the risk of liver injury with DOACs compared with warfarin [34]. These results suggest that DOACs may be suitable alternatives to warfarin in patients with liver disease.

The approach for management and monitoring bleeding risks in people who are taking antithrombotic medicines should be, in principle, the same in patients with and without liver disease. However, patients with liver disease may benefit from additional risk-benefit assessments using liver function tests, screening for ongoing alcohol use, measuring coagulation profile and platelet count before initiation and during treatment at more frequent intervals. The American Association for the Study of Liver disease also recommends screening for varices before the initiation of anticoagulants [35]. Patients with liver disease should be informed of potential benefits and risks of antithrombotic therapy (especially in patients with active coagulopathy) and be included in decision-making on the selection of specific medicines to promote adherence while minimising risks. Multidisciplinary team meetings between hepatologists and cardiologists may be required to discuss treatment options and explore additional strategies on reducing risk.

### 4.2 Working with patients to improve adherence

Our study considered adherence and persistence in combination to return insights that may allow personalised approaches for supporting adherence. Our results suggest that there are significant geographical variations in the prescribing prevalence, adherence to and persistence with antithrombotic medications. These results suggest that there might be regional variations in risk factors influencing medication adherence, differences in how individuals access health services and differences in risk awareness across communities. This work may pave the way for health and care services to create local solutions targeting specific populations. We observed that in both anticoagulant and antiplatelet therapy, ‘non-adherent, persistence’ is common among people with and without liver disease. Notwithstanding the high economic costs of wasted medicines, non-adherence may limit the efficacy of drugs and could result in health deterioration and subsequent knock-on effects of poor health. Dosing frequency is a common factor that affects adherence. Patients were found to be more adherent with once-a-day dosing frequency compared with more frequent medication regimens [36]. Patients with chronic liver disease may experience polypharmacy due to multimorbidity, which could increase the risk of non-adherence. Individuals with viral hepatitis are required to take antiviral medications daily and many people with NAFLD also have type 2 diabetes, hypertension and hypercholesterolaemia which require pharmacological interventions. Multi-drug regimens can increase non-adherence either because of the higher number of medicines that can be potentially missed or the presence of complex dosing instructions [37]. Hepatic encephalopathy is a common complication in patients with severe liver disease. Patients with advanced liver disease are often frail and having strong support from social and familial networks may help promote adherence. Other non-patient-centric factors of polypharmacy and non-adherence include cost and fragmentation of care, where patients are seen by different specialists who may not be in communication with each other. Patients with liver disease may have an increased need for ongoing safety monitoring and may have experienced negative effects from other medications - both factors could further promote non-adherence.

Issues leading to non-adherence will be different for each patient. Tackling non-adherence begins with understanding patients’ views of medicines to help them make informed decisions about appropriate use. Patients with liver disease may experience unintentional non-adherence due to reasons beyond their control. Chronic HCV infection is associated with drug use and deprivation and may affect patients’ ability to adhere to the agreed treatment. We should not consider non-adherence as the patients’ problem since adherence is dependent on an agreement between patients and their doctors on medication recommendations [38]. Addressing non-adherence requires an understanding of patients' beliefs and concerns about medicines and reasons why they might want to reduce the number of medications they take. Patients might be afraid to express their concerns about medicines, hence, overcoming non-adherence may require frequent conversations about adherence in a non-accusatory way to identify opportunities for intervention.

### 4.3 Overall recommendations

Based on our results, once a day regimen might improve adherence and persistence especially in patients who need to take multiple medications. Furthermore, DOACs have a shorter half-life and are less reliant on hepatic clearance compared with warfarin, which may be more suitable for people with liver disease [15,39]. However, DOACs still require hepatorenal clearance and cytochrome P450 metabolism (activity is reduced in diseased liver), meaning that caution is recommended in patients with liver disease with co-existing kidney diseases. Certain DOACs such as rivaroxaban and apixaban have high plasma protein binding capacity which may cause increased free drug levels when albumin synthesis in the liver is impaired [40]. Selecting DOACs for which antidotes are available may help mitigate against potential complications. For example, Idarucizumab (Praxbind) is approved by the European Medicines Agency to neutralise the effects of dabigatran. Andexanet alfa (Ondexxya) is approved for use as an antidote against apixaban and rivaroxaban.

### 4.4 Strength and limitations of the study

Our analyses have several important strengths. To the best of our knowledge, this is the first study that examined prescribing prevalence, adherence, persistence (and geographical variations), risk of non-adherence and non-persistence and effects of adherence on bleeding and stroke for anticoagulant and antiplatelet medicines in patients with and without liver disease. Second, is the use of population health records for estimating prescribing prevalence of anticoagulant and antiplatelet medications involving six chronic liver conditions, including less prevalent conditions such as autoimmune liver disease. Third, we analysed five types of anticoagulants and five types of antiplatelets that included new generation medicines. Fourth, we considered the relationship between adherence and persistence in combination, at 6 and 12 months, in patients with and without liver disease. Fifth, we harnessed linked records from primary and secondary care, which allowed more accurate case ascertainment for diagnoses, comorbidities, bleeding and stroke outcomes.

We acknowledge several limitations in our analyses. There are numerous methods for measuring adherence. We have employed previously validated methods to estimate adherence from prescription data based on the proportion of days covered [27,29,41,42]. Missing data is common in electronic health records, and we were unable to include individuals with insufficient follow-up. There may be residual unmeasured confounding as with all observational studies. A relatively low number of patients with liver disease were analysed for DOACs. Our analyses are restricted to drug-naïve patients to minimise bias associated with previous antithrombotic use; however, we were unable to exclude over-the-counter aspirin use. We also did not evaluate subsequent medicine use in non-naïve patients.

This study demonstrates the importance of considering adherence and persistence together in the management of antithrombotic therapy in patients with liver disease. Our work may help overcome the issue of limited randomised trial evidence on the safety and efficacy of these drugs in people who are contraindicated. Results may inform medicines optimisation strategies in these high-risk patients. We found that patients with liver disease are more adherent to certain medications. Compared with patients without liver disease, patients with liver disease who stopped taking antiplatelets had a higher risk of stroke, however, adherence to antiplatelets was associated with increased bleeding risk. We considered challenges and opportunities for addressing non-adherence, which emphasise the need for involving patients in shared decision-making. Non-adherence is a complex issue; our work provides a much-needed evidence-base that may encourage patients with contraindications to antithrombotic therapy to be involved in discussions with their doctors on benefits and risks.

## Contributors

Research question: WHC and AGL

Funding: AGL

Study design and analysis plan: WHC and AGL

Preparation of data: WHC and AGL

Statistical analysis: WHC and AGL

Generation of scripts for plotting maps: SM

Generation of prescription phenotypes: YYT

Drafting initial and final versions of manuscript: WHC and AGL

Critical review of early and final versions of manuscript: All authors

WHC and AGL have directly accessed and verified the underlying data reported in the manuscript.

## Data availability statement

The data used in this study are available on successful ethics application to the Clinical Practice Research Datalink (CPRD). All summarised data and results are made available as supplementary materials.

## Declaration of Interests

None declared.

## Appendix Supplementary materials

Image, application 1

Figure S1. Flow diagram of study population for prescribing prevalence and adherence and persistence analyses. (A) Analysis of anticoagulants in patients with liver disease. (B) Analysis of anticoagulants in patients without liver disease. (C) Analysis of antiplatelets in patients with liver disease. (D) Analysis of antiplatelets in patients without liver disease. CLD: chronic liver disease; AF: atrial fibrillation; MI: myocardial infarction; PAD: peripheral arterial disease; TIA: transient ischaemic attack; UA: unstable angina. Table S1. Baseline table showing the characteristics of patients at the time of first anticoagulant or antiplatelet prescription on or after diagnosis of a cardiovascular indication in patients with chronic liver disease. Table S2. Baseline table showing the characteristics of patients at the time of first anticoagulant or antiplatelet prescription on or after diagnosis of a cardiovascular indication in patients without chronic liver disease. Table S3. Details on the prescribing prevalence of antithrombotic medications in patients with or without chronic liver disease (as shown in Figure 1). Table S4. Details on the proportion of patients who were adherent to antithrombotic medications at 12 months, in patients with or without chronic liver disease (as shown in Figure 2). Patients having proportion of days covered (PDC) of > 80% were considered adherent. Table S5. Details on the proportion of patients who were persistent to antithrombotic medications at 12 months, in patients with or without chronic liver disease (as shown in Figure 3). Table S6. Summary data for outcomes of interests pertaining to Table 1 (adherence) and Table 2 (persistence). Table S7. Summary data for outcomes of interests pertaining to Table 4 (stroke and bleeding) in patients without chronic liver disease. Table S8. Summary data for outcomes of interests pertaining to Table 5 (comparison of stroke and bleeding risk between patients with and without chronic liver disease (CLD)). Image, application 2

## Footnotes

Supplementary material associated with this article can be found, in the online version, at doi:10.1016/j.lanepe.2021.100222.
