# Supplementary material for: Antithrombotic therapy in patients with liver disease: population-based insights on variations in prescribing trends, adherence, persistence and impact on stroke and bleeding
Source: Lancet Reg Health Eur. 2021 Sep 8;10:100222. doi: 10.1016/j.lanepe.2021.100222 (PMC8589727; doi:10.1016/j.lanepe.2021.100222)
Supplement: Supplementary file 1 [file mmc1.docx]

**Supplementary appendix**

***Regional variations in prescribing prevalence***

We analysed regional variations in prescribing prevalence and generated maps of variation. Prescribing prevalence was estimated per 100 persons with CVD indications. For anticoagulant prescribing, the South East Coast region had the highest prevalence in both groups: with liver disease (28.5% [140/492]; CI: 24.5 - 32.4%) and without liver disease (39.2% [17,806/45,416]; 38.8 - 39.7%) (Figure 1). This was followed by the North West region where prescribing prevalence was 24.8% [189/763] and 35.1% [16,269/46,347] in patients with or without liver disease, respectively. Patients with liver disease living in the North East had the lowest prescribing prevalence for anticoagulants at 8.0% [9/112]. In individuals without liver disease, the lowest prevalence was observed in East Midlands at 24.4% [2,707/11,097].

When considering each liver condition separately, patients with ALD (21.8% [41/188]), cirrhosis (21.9% [49/224]) and NAFLD (35.2% [74/210]) living in the South East Coast region had the highest prescribing prevalence for anticoagulants. For people with HCV and autoimmune liver disease, individuals living in the North West region had the highest rate of prescribing at 23.1% [9/39] and 30.2% [16/53], respectively. For HBV, the highest rate of prescribing was observed in the South Central region (44.7% [17/38]) (Figure 1). The North East region had the lowest rate of anticoagulant prescribing in patients with ALD (4.2% [2/48]) and cirrhosis (6.9% [4/58]) and the second-lowest rate in patients with autoimmune liver disease (11.8% [2/17]) and NAFLD (8.1% [3/37]) (Figure 1).

For antiplatelets, prescribing prevalence was the highest in the North West region (73.9% [44,547/60,318]), followed by the North East region (73.7% [6,248/8,472]) in individuals without liver disease (Figure 1). A similar trend of high prescribing prevalence was observed in North West (60.1% [555/924]; prevalence for London was 60.2% [327/543]) and North East (57.5% [65/113]) regions in people with liver disease. In contrast, East Midlands had the lowest prevalence in both groups: with liver disease (43.8% [53/121]) and without liver disease (65.5% [7,879/12,033]).

An exploration of regional variations in antiplatelet prescribing across liver conditions revealed that London came out on top as the region with the highest prevalence in patients with ALD (61.8% [141/228]) and cirrhosis (59.7% [132/221]). In patients with autoimmune liver disease (69.7% [23/33]), HBV (71.9% [23/32]) or NAFLD (57.6% [121/210]), London was the second-highest region. In patients with HCV, individuals living in the South Central region (51.3% [20/39]) had the highest prescribing prevalence, while those living in London (45.2% [33/73]) had the third-highest prevalence (Figure 1). In contrast, prescribing prevalence for antiplatelets was the lowest in East Midlands in patients with ALD (40.4% [21/52]), autoimmune liver disease (50.0% [6/12]) or HBV (20.0% [1/5]) and the second-lowest in patients with cirrhosis (47.5% [28/59]) or NAFLD (42.9% [18/42]) (Figure 1). Overall, patients with liver disease had a lower prescribing prevalence for anticoagulants and antiplatelets compared with individuals without liver disease. Regional variations in prescribing prevalence were observed when comparing liver diseases.

***Regional variations in adherence***

In patients with liver disease, adherence to any anticoagulants at 12 months ranged from 20.0% [2/10] (East Midlands) to 36.3% [29/80] (South West). In patients without liver disease, the range of adherence to anticoagulants was from 22.4% [1,888/8,410] (London) to 36.2% [5,147/14,233] (North West) (Figure 2). When considering specific anticoagulants, adherence to apixaban was the highest in London (53.3% [8/15]) in patients with liver disease while in patients without liver disease, adherence was the highest in East Midlands (60.0% [3/5]). For rivaroxaban, adherence ranged from 25.0% [1/4] (East of England) to 75.0% [15/20] (London) in patients with liver disease. In individuals without liver disease, adherence to rivaroxaban was the lowest in East Midlands (15.4% [2/13]) and highest in West Midlands (46.1% [399/866]). Adherence to warfarin ranged from 19.1% [9/47] (South East Coast) to 39.2% [20/51] (South Central) in patients with liver disease. In contrast, adherence to warfarin in individuals without liver disease was the lowest in London (19.6% [1,404/7,158]) and the highest in the North East (34.1% [566/1,660]) (Figure 2).

We explored adherence to any antiplatelets and observed that in patients with liver disease, adherence ranged from 29.9% [79/264] (London) to 59.6% [34/57] (Yorkshire and the Humber). In patients without liver disease, adherence to any antiplatelets was also the lowest in London (26.2% [5,615/21,414]) and the highest in Yorkshire and the Humber (51.2% [5,453/10,652]) (Figure 2). When exploring specific antiplatelets, adherence to aspirin was the highest in Yorkshire and the Humber in people with (63.6% [28/44]) and without liver disease (48.8% [4,663/9,558]). For clopidogrel, adherence in people with liver disease ranged from 27.0% [33/122] (London) to 57.2% [123/215] (North West). In people without liver disease, the lowest adherence to clopidogrel was also observed in London (32.0% [2,535/7,933]), but the highest adherence was observed in Yorkshire and the Humber (49.7% [1,457/2,930]). For dipyridamole, adherence in patients with liver disease ranged from 9.1% [1/11] (London) to 50.0% [12/24] (North West), while in patients without liver disease, adherence ranged from 34.2% [464/1,356] (London) to 44.4% [460/1,036] (Yorkshire and the Humber) (Figure 2).

***Regional variations in persistence***

Persistence with any anticoagulants at 12 months ranged from 50.0% [5/10] (East Midlands) to 74.7% [71/95] (London) in patients with liver disease. Persistence in patients without liver disease ranged from 59.8% [6,651/11,114] (South Central) to 69.5% [9,746/14,024] (North West) (Figure 3). For apixaban, persistence was the highest in the North West region in both with (81.1% [30/37]) and without liver disease (76.6% [1,264/1,651]) groups. Persistence with rivaroxaban ranged from 42.9% [3/7] (South Central) to 95.0% [19/20] (London) in patients with liver disease, while in patients without liver disease, persistence ranged from 30.8% [4/13] (East Midlands) to 73.7% [2,342/3,179] (South East Coast). For warfarin, the highest persistence was observed in East of England (75.6% [31/41]) and the lowest in East Midlands (50.0% [5/10]) in patients with liver disease. In contrast, in people without liver disease, London had the lowest persistence (58.7% [4,199/7,158]) while people in the North East region had the highest persistence (69.3% [1,151/1,660]) (Figure 3).

Persistence with any antiplatelets at 12 months in patients with liver disease ranged from 60.5% [26/43] (East Midlands) to 78.2% [43/55] (Yorkshire and the Humber). In patients without liver disease, London had the lowest persistence (62.6% [12,771/20,396]) while Yorkshire and the Humber had the highest persistence (73.4% [7,503/10,228]) (Figure 3). For aspirin, the highest persistence was observed in Yorkshire and the Humber in patients with (79.5% [35/44]) and without (73.1% [6,985/9,558]) liver disease. Persistence with clopidogrel in patients with liver disease ranged from 64.3% [9/14] (East Midlands) to 89.3% [25/28] (Yorkshire and the Humber). In patients without liver disease, persistence ranged from 70.4% [1,167/1,657] (East Midlands) to 77.8% [1,277/1,642] (North East). For dipyridamole, patients with liver disease living in the South West had the lowest persistence (58.3% [7/12]). In patients without liver disease, persistence with dipyridamole ranged from 70.6% [958/1,356] (London) to 74.9% [776/1,036] (Yorkshire and the Humber) (Figure 3).
