# Supplementary material for: Antithrombotic therapy in patients with liver disease: population-based insights on variations in prescribing trends, adherence, persistence and impact on stroke and bleeding
Source: Lancet Reg Health Eur. 2021 Sep 8;10:100222. doi: 10.1016/j.lanepe.2021.100222 (PMC8589727; doi:10.1016/j.lanepe.2021.100222)
Supplement: Supplementary file 2 — Figure S1. Flow diagram of study population for prescribing prevalence and adherence and persistence analyses. (A) Analysis of anticoagulants in patients with liver disease. (B) Analysis of anticoagulants in patients without liver disease. (C) Analysis of antiplatelets in patients with liver disease. (D) Analysis of antiplatelets in patients without liver disease. CLD: chronic liver disease; AF: atrial fibrillation; MI: myocardial infarction; PAD: peripheral arterial disease; TIA: transient ischaemic attack; UA: unstable angina. Table S1. Baseline table showing the characteristics of patients at the time of first anticoagulant or antiplatelet prescription on or after diagnosis of a cardiovascular indication in patients with chronic liver disease. Table S2. Baseline table showing the characteristics of patients at the time of first anticoagulant or antiplatelet prescription on or after diagnosis of a cardiovascular indication in patients without chronic liver disease. Table S3. Details on the prescribing prevalence of antithrombotic medications in patients with or without chronic liver disease (as shown in Figure 1). Table S4. Details on the proportion of patients who were adherent to antithrombotic medications at 12 months, in patients with or without chronic liver disease (as shown in Figure 2). Patients having proportion of days covered (PDC) of > 80% were considered adherent. Table S5. Details on the proportion of patients who were persistent to antithrombotic medications at 12 months, in patients with or without chronic liver disease (as shown in Figure 3). Table S6. Summary data for outcomes of interests pertaining to Table 1 (adherence) and Table 2 (persistence). Table S7. Summary data for outcomes of interests pertaining to Table 4 (stroke and bleeding) in patients without chronic liver disease. Table S8. Summary data for outcomes of interests pertaining to Table 5 (comparison of stroke and bleeding risk between patients with and without chronic liver disease [file mmc2.pdf]

Supplementary file for

Antithrombotic therapy in patients with liver disease: Population-based insights on variations in prescribing trends, adherence, persistence and impact on stroke and bleeding

Wai Hoong Chang, Stefanie H. Mueller, Yen Yi Tan, Alvina G. Lai

Figure S1. Flow diagram of study population for prescribing prevalence and adherence and persistence analyses. (A) Analysis of anticoagulants in patients with liver disease. (B) Analysis of anticoagulants in patients without liver disease. (C) Analysis of antiplatelets in patients with liver disease. (D) Analysis of antiplatelets in patients without liver disease. CLD: chronic liver disease; AF: atrial fibrillation; MI: myocardial infarction; PAD: peripheral arterial disease; TIA: transient ischaemic attack; UA: unstable angina.

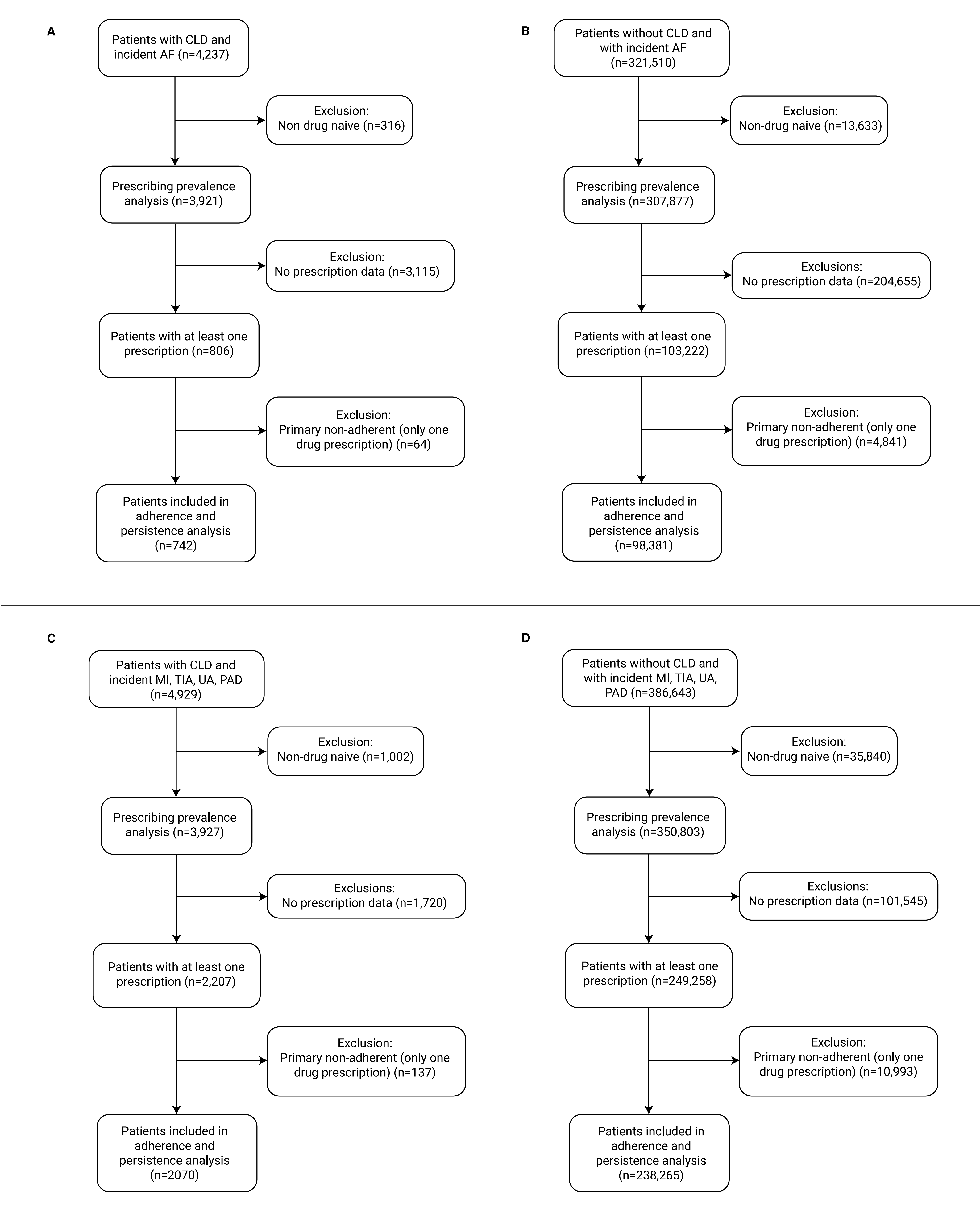

Table S1. Baseline table showing the characteristics of patients at the time of first anticoagulant or antiplatelet prescription on or after diagnosis of a cardiovascular indication in patients with chronic liver disease.

	Anticoagulants				Antiplatelets		
	**Men**	**Women**	**Overall**		**Men**	**Women**	**Overall**
**n**	500	306	806	**n**	1440	767	2207
**Age at first prescription (mean (SD))**	69.1 (10.47)	73.6 (10.67)	70.8 (10.76)	**Age at first prescription (mean (SD))**	64.0 (11.06)	68.8 (11.63)	65.7 (11.49)
**Age group (%)**				**Age group (%)**			
Age 30 - 49	22 (4.4)	11 (3.6)	33 (4.1)	Age 30 - 49	141 (9.8)	54 (7.0)	195 (8.8)
Age 50 - 59	69 (13.8)	21 (6.9)	90 (11.2)	Age 50 - 59	382 (26.5)	120 (15.6)	502 (22.7)
Age 60 - 69	157 (31.4)	58 (19.0)	215 (26.7)	Age 60 - 69	480 (33.3)	204 (26.6)	684 (31.0)
Age 70 - 79	175 (35.0)	130 (42.5)	305 (37.8)	Age 70 - 79	319 (22.2)	255 (33.2)	574 (26.0)
Age 80 & above	77 (15.4)	86 (28.1)	163 (20.2)	Age 80 & above	118 (8.2)	134 (17.5)	252 (11.4)
**Practice region (%)**				**Practice region (%)**			
North East	4 (0.8)	5 (1.6)	9 (1.1)	North East	43 (3.0)	22 (2.9)	65 (2.9)
North West	116 (23.2)	73 (23.9)	189 (23.4)	North West	357 (24.8)	198 (25.8)	555 (25.1)
Yorkshire & The Humber	12 (2.4)	6 (2.0)	18 (2.2)	Yorkshire & The Humber	45 (3.1)	26 (3.4)	71 (3.2)
East Midlands	6 (1.2)	7 (2.3)	13 (1.6)	East Midlands	25 (1.7)	28 (3.7)	53 (2.4)
West Midlands	49 (9.8)	28 (9.2)	77 (9.6)	West Midlands	170 (11.8)	74 (9.6)	244 (11.1)
East of England	30 (6.0)	29 (9.5)	59 (7.3)	East of England	76 (5.3)	57 (7.4)	133 (6.0)
South West	55 (11.0)	46 (15.0)	101 (12.5)	South West	180 (12.5)	105 (13.7)	285 (12.9)
South Central	56 (11.2)	28 (9.2)	84 (10.4)	South Central	171 (11.9)	81 (10.6)	252 (11.4)
London	86 (17.2)	30 (9.8)	116 (14.4)	London	233 (16.2)	94 (12.3)	327 (14.8)
South East Coast	86 (17.2)	54 (17.6)	140 (17.4)	South East Coast	140 (9.7)	82 (10.7)	222 (10.1)
**Comorbidities (%)**				**Comorbidities (%)**			
Chronic kidney disease	114 (22.8)	110 (35.9)	224 (27.8)	Chronic kidney disease	234 (16.2)	199 (25.9)	433 (19.6)
Ascites	87 (17.4)	36 (11.8)	123 (15.3)	Ascites	239 (16.6)	118 (15.4)	357 (16.2)
Cirrhosis	202 (40.4)	120 (39.2)	322 (40.0)	Cirrhosis	584 (40.6)	302 (39.4)	886 (40.1)
Hepatic encephalopathy	36 (7.2)	8 (2.6)	44 (5.5)	Hepatic encephalopathy	111 (7.7)	56 (7.3)	167 (7.6)
Varices	47 (9.4)	32 (10.5)	79 (9.8)	Varices	183 (12.7)	81 (10.6)	264 (12.0)
Proton pump inhibitor use	279 (55.8)	174 (56.9)	453 (56.2)	Proton pump inhibitor use	875 (60.8)	511 (66.6)	1386 (62.8)
**Liver disease (%)**				**Liver disease (%)**			
Alcoholic liver disease	219 (43.8)	56 (18.3)	275 (34.1)	Alcoholic liver disease	687 (47.7)	212 (27.6)	899 (40.7)
Autoimmune liver disease	21 (4.2)	67 (21.9)	88 (10.9)	Autoimmune liver disease	56 (3.9)	139 (18.1)	195 (8.8)
Cirrhosis	202 (40.4)	120 (39.2)	322 (40.0)	Cirrhosis	584 (40.6)	302 (39.4)	886 (40.1)
Chronic hepatitis B infection	41 (8.2)	17 (5.6)	58 (7.2)	Chronic hepatitis B infection	108 (7.5)	37 (4.8)	145 (6.6)
Chronic hepatitis C infection	26 (5.2)	11 (3.6)	37 (4.6)	Chronic hepatitis C infection	111 (7.7)	34 (4.4)	145 (6.6)
Non-alcoholic fatty liver disease	204 (40.8)	127 (41.5)	331 (41.1)	Non-alcoholic fatty liver disease	504 (35.0)	298 (38.9)	802 (36.3)
**Child-Pugh score (%)**				**Child-Pugh score (%)**			
Class A (score 5-6)	295 (59.0)	208 (68.0)	503 (62.4)	Class A (score 5-6)	939 (65.2)	530 (69.1)	1469 (66.6)
Class B (score 7-9)	199 (39.8)	96 (31.4)	295 (36.6)	Class B (score 7-9)	476 (33.1)	224 (29.2)	700 (31.7)
Class C (score 10-15)	6 (1.2)	2 (0.7)	8 (1.0)	Class C (score 10-15)	25 (1.7)	13 (1.7)	38 (1.7)
**FIB-4 score (%)**				**FIB-4 score (%)**			
< 1.45	213 (42.6)	132 (43.1)	345 (42.8)	< 1.45	840 (58.3)	400 (52.2)	1240 (56.2)
1.45-3.25	239 (47.8)	149 (48.7)	388 (48.1)	1.45-3.25	486 (33.8)	301 (39.2)	787 (35.7)
>3.25	48 (9.6)	25 (8.2)	73 (9.1)	>3.25	114 (7.9)	66 (8.6)	180 (8.2)
**Medication type (%)**				**Medication type (%)**			
Warfarin	360 (72.0)	208 (68.0)	568 (70.5)	Aspirin	1239 (86.0)	636 (82.9)	1875 (85.0)
Apixaban	82 (16.4)	61 (19.9)	143 (17.7)	Clopidogrel	690 (47.9)	372 (48.5)	1062 (48.1)
Rivaroxaban	87 (17.4)	61 (19.9)	148 (18.4)	Dipyridamole	99 (6.9)	48 (6.3)	147 (6.7)
**CHA^{2}DS^2 VASc score (relevant to anticoagulants only) (%)**							
0-1	100 (20.0)	14 (4.6)	114 (14.1)				
2	107 (21.4)	22 (7.2)	129 (16.0)				
3-4	182 (36.4)	130 (42.5)	312 (38.7)				
5-9	111 (22.2)	140 (45.8)	251 (31.1)				
**Warfarin anticoagulant time in therapeutic range (TTR) (%)**							
< 60%	113 (54.3)	70 (51.1)	183 (53.0)				
60-80%	59 (28.4)	48 (35.0)	107 (31.0)				
> 80%	36 (17.3)	19 (13.9)	55 (15.9)				

Table S2. Baseline table showing the characteristics of patients at the time of first anticoagulant or antiplatelet prescription on or after diagnosis of a cardiovascular indication in patients without chronic liver disease.

	Anticoagulants				Antiplatelets		
	Men	Women	Overall		Men	Women	Overall
n	57709	45513	103222	n	143453	105805	249258
**Age at first prescription (mean (SD))**	72.5 (10.98)	77.2 (9.94)	74.6 (10.79)	**Age at first prescription (mean (SD))**	68.2 (11.98)	74.6 (12.15)	70.9 (12.47)
**Age group (%)**				**Age group (%)**			
Age 30 - 49	2057 (3.6)	644 (1.4)	2701 (2.6)	Age 30 - 49	10463 (7.3)	3868 (3.7)	14331 (5.7)
Age 50 - 59	5297 (9.2)	1915 (4.2)	7212 (7.0)	Age 50 - 59	24835 (17.3)	9069 (8.6)	33904 (13.6)
Age 60 - 69	13857 (24.0)	6885 (15.1)	20742 (20.1)	Age 60 - 69	42806 (29.8)	21190 (20.0)	63996 (25.7)
Age 70 - 79	21235 (36.8)	16509 (36.3)	37744 (36.6)	Age 70 - 79	40464 (28.2)	32695 (30.9)	73159 (29.4)
Age 80 & above	15263 (26.4)	19560 (43.0)	34823 (33.7)	Age 80 & above	24885 (17.3)	38983 (36.8)	63868 (25.6)
**Practice region (%)**				**Practice region (%)**			
North East	1115 (1.9)	915 (2.0)	2030 (2.0)	North East	3579 (2.5)	2669 (2.5)	6248 (2.5)
North West	9044 (15.7)	7225 (15.9)	16269 (15.8)	North West	25296 (17.6)	19251 (18.2)	44547 (17.9)
Yorkshire & The Humber	2117 (3.7)	1673 (3.7)	3790 (3.7)	Yorkshire & The Humber	6643 (4.6)	5182 (4.9)	11825 (4.7)
East Midlands	1532 (2.7)	1175 (2.6)	2707 (2.6)	East Midlands	4566 (3.2)	3313 (3.1)	7879 (3.2)
West Midlands	7134 (12.4)	5649 (12.4)	12783 (12.4)	West Midlands	17714 (12.3)	13277 (12.5)	30991 (12.4)
East of England	5592 (9.7)	4417 (9.7)	10009 (9.7)	East of England	14590 (10.2)	10530 (10.0)	25120 (10.1)
South West	8580 (14.9)	6804 (14.9)	15384 (14.9)	South West	20303 (14.2)	14955 (14.1)	35258 (14.1)
South Central	7272 (12.6)	5522 (12.1)	12794 (12.4)	South Central	17415 (12.1)	12571 (11.9)	29986 (12.0)
London	5304 (9.2)	4346 (9.5)	9650 (9.3)	London	13887 (9.7)	10286 (9.7)	24173 (9.7)
South East Coast	10019 (17.4)	7787 (17.1)	17806 (17.3)	South East Coast	19460 (13.6)	13771 (13.0)	33231 (13.3)
**Medication type (%)**				**Medication type (%)**			
Warfarin	49288 (85.4)	38503 (84.6)	87791 (85.1)	Aspirin	134229 (93.6)	96680 (91.4)	230909 (92.6)
Apixaban	5348 (9.3)	4680 (10.3)	10028 (9.7)	Clopidogrel	52774 (36.8)	34712 (32.8)	87486 (35.1)
Rivaroxaban	6330 (11.0)	5270 (11.6)	11600 (11.2)	Dipyridamole	12382 (8.6)	10590 (10.0)	22972 (9.2)
**CHA^2DS^2 VASc score (relevant to anticoagulants only) (%)**							
0-1	12742 (22.1)	1836 (4.0)	14578 (14.1)				
2	12005 (20.8)	4064 (8.9)	16069 (15.6)				
3-4	22223 (38.5)	20618 (45.3)	42841 (41.5)				
5-9	10739 (18.6)	18995 (41.7)	29734 (28.8)				

Table S3. Details on the prescribing prevalence of antithrombotic medications in patients with or without chronic liver disease in England (as shown in Figure 1).

Liver disease	Denominator	Numerator	Prevalence	Lower CI	Upper CI	Antithrombotic type
Alcoholic liver disease	1629	275	16.9	15.1	18.7	Anticoagulants
Autoimmune liver disease	364	88	24.2	19.8	28.6	Anticoagulants
Cirrhosis	1827	322	17.6	15.9	19.4	Anticoagulants
Chronic HBV infection	194	58	29.9	23.5	36.3	Anticoagulants
Chronic HCV infection	228	37	16.2	11.4	21.0	Anticoagulants
Non-alcoholic fatty liver disease	1474	331	22.5	20.3	24.6	Anticoagulants
Any liver disease	3921	806	20.6	19.3	21.8	Anticoagulants
No liver disease	307877	103222	33.5	33.4	33.7	Anticoagulants
Alcoholic liver disease	1639	899	54.9	52.4	57.3	Antiplatelets
Autoimmune liver disease	317	195	61.5	56.2	66.9	Antiplatelets
Cirrhosis	1592	886	55.7	53.2	58.1	Antiplatelets
Chronic HBV infection	253	145	57.3	51.2	63.4	Antiplatelets
Chronic HCV infection	374	145	38.8	33.8	43.7	Antiplatelets
Non-alcoholic fatty liver disease	1424	802	56.3	53.7	58.9	Antiplatelets
Any liver disease	3927	2207	56.2	54.6	57.8	Antiplatelets
No liver disease	350803	249258	71.1	70.9	71.2	Antiplatelets

Table S4. Details on the proportion of patients who were adherent to antithrombotic medications at 12 months, in patients with or without chronic liver disease (as shown in Figure 2). Patients having proportion of days covered (PDC) of > 80% were considered adherent.

Liver disease	Denominator	Numerator	Proportion adherent	Lower CI	Upper CI	Antithrombotic type
Alcoholic liver disease	204	80	39.2	32.5	45.9	Antiplatelets
Autoimmune liver disease	50	23	46.0	32.2	59.8	Antiplatelets
Cirrhosis	200	78	39.0	32.2	45.8	Antiplatelets
Chronic HBV infection	26	8	30.8	13.0	48.5	Antiplatelets
Chronic HCV infection	26	13	50.0	30.8	69.2	Antiplatelets
Non-alcoholic fatty liver disease	146	64	43.8	35.8	51.9	Antiplatelets
Any liver disease	1818	743	40.9	38.6	43.1	Antiplatelets
No liver disease	223154	76834	34.4	34.2	34.6	Antiplatelets
Alcoholic liver disease	214	64	29.9	23.8	36.0	Anticoagulants
Autoimmune liver disease	72	27	37.5	26.3	48.7	Anticoagulants
Cirrhosis	255	79	31.0	25.3	36.7	Anticoagulants
Chronic HBV infection	46	16	34.8	21.0	48.5	Anticoagulants
Chronic HCV infection	32	8	25.0	10.0	40.0	Anticoagulants
Non-alcoholic fatty liver disease	248	93	37.5	31.5	43.5	Anticoagulants
Any liver disease	628	208	33.1	29.4	36.8	Anticoagulants
No liver disease	90569	26615	29.4	29.1	29.7	Anticoagulants
No liver disease	197656	62276	31.5	31.3	31.7	Aspirin
No liver disease	72016	27870	38.7	38.3	39.1	Clopidogrel
No liver disease	17681	6585	37.2	36.5	38.0	Dipyridamole
Any liver disease	1482	540	36.4	34.0	38.9	Aspirin
Any liver disease	810	340	42.0	38.6	45.4	Clopidogrel
Any liver disease	103	32	31.1	22.1	40.0	Dipyridamole
No liver disease	7584	3544	46.7	45.6	47.9	Apixaban
No liver disease	9135	3828	41.9	40.9	42.9	Rivaroxaban
No liver disease	77370	20302	26.2	25.9	26.6	Warfarin
Any liver disease	103	44	42.7	33.2	52.3	Apixaban
Any liver disease	101	52	51.5	41.7	61.2	Rivaroxaban
Any liver disease	453	125	27.6	23.5	31.7	Warfarin

Table S5. Details on the proportion of patients who were persistent to antithrombotic medications at 12 months, in patients with or without chronic liver disease (as shown in Figure 3).

Liver disease	Denominator	Numerator	Proportion persistent	Lower CI	Upper CI	Antithrombotic type
With liver disease	615	402	65.4	61.6	69.1	Any anticoagulant
Without liver disease	89022	57642	64.8	64.4	65.1	Any anticoagulant
With liver disease	1718	1175	68.4	66.2	70.6	Any antiplatelet
Without liver disease	212448	142855	67.2	67.0	67.4	Any antiplatelet
Without liver disease	77370	49687	64.2	63.9	64.6	Warfarin
With liver disease	453	295	65.1	60.7	69.5	Warfarin
Without liver disease	7584	5334	70.3	69.3	71.4	Apixaban
With liver disease	103	69	67.0	57.9	76.1	Apixaban
Without liver disease	9135	6217	68.1	67.1	69.0	Rivaroxaban
With liver disease	101	75	74.3	65.7	82.8	Rivaroxaban
Without liver disease	197656	131953	66.8	66.6	67.0	Aspirin
With liver disease	1482	1018	68.7	66.3	71.1	Aspirin
Without liver disease	17681	12904	73.0	72.3	73.6	Dipyridamole
With liver disease	103	77	74.8	66.4	83.1	Dipyridamole
Without liver disease	72016	53298	74.0	73.7	74.3	Clopidogrel
With liver disease	810	593	73.2	70.2	76.3	Clopidogrel

Table S6. Summary data for outcomes of interests pertaining to Table 1 (adherence) and Table 2 (persistence).

Outcome	Adherent at 6 months	Non-adherent at 6 months	Adherent at 12 months	Non-adherent at 12 months	Persistent at 6 months	Non-persistent at 6 months	Persistent at 12 months	Non-persistent at 12 months
**n (denominator)**	1182	1463	951	1495	2121	524	1636	810
**Female (%)**	462 (39.1)	472 (32.3)	367 (38.6)	496 (33.2)	758 (35.7)	176 (33.6)	587 (35.9)	276 (34.1)
**Age (%)**								
30 - 49	87 (7.4)	116 (7.9)	66 (6.9)	132 (8.8)	158 (7.4)	45 (8.6)	128 (7.8)	70 (8.6)
50 - 59	220 (18.6)	311 (21.3)	191 (20.1)	307 (20.5)	424 (20.0)	107 (20.4)	344 (21.0)	154 (19.0)
60 - 69	351 (29.7)	459 (31.4)	288 (30.3)	471 (31.5)	653 (30.8)	157 (30.0)	511 (31.2)	248 (30.6)
70 - 79	328 (27.7)	429 (29.3)	260 (27.3)	438 (29.3)	607 (28.6)	150 (28.6)	463 (28.3)	235 (29.0)
80 above	196 (16.6)	148 (10.1)	146 (15.4)	147 (9.8)	279 (13.2)	65 (12.4)	190 (11.6)	103 (12.7)
**Comorbidities (%)**								
Chronic kidney disease	260 (22.0)	256 (17.5)	204 (21.5)	258 (17.3)	420 (19.8)	96 (18.3)	314 (19.2)	148 (18.3)
Ascites	174 (14.7)	226 (15.4)	133 (14.0)	231 (15.5)	309 (14.6)	91 (17.4)	231 (14.1)	133 (16.4)
Cirrhosis	477 (40.4)	654 (44.7)	374 (39.3)	667 (44.6)	884 (41.7)	247 (47.1)	678 (41.4)	363 (44.8)
Hepatic encephalopathy	89 (7.5)	91 (6.2)	64 (6.7)	98 (6.6)	147 (6.9)	33 (6.3)	105 (6.4)	57 (7.0)
Varices	112 (9.5)	173 (11.8)	87 (9.1)	183 (12.2)	221 (10.4)	64 (12.2)	166 (10.1)	104 (12.8)
Proton pump inhibitor use	760 (64.3)	840 (57.4)	598 (62.9)	871 (58.3)	1305 (61.5)	295 (56.3)	1002 (61.2)	467 (57.7)
**CHA^2DS^2 VASc score (%)**								
0-1	141 (11.9)	205 (14.0)	118 (12.4)	212 (14.2)	257 (12.1)	89 (17.0)	202 (12.3)	128 (15.8)
2	186 (15.7)	267 (18.3)	155 (16.3)	274 (18.3)	373 (17.6)	80 (15.3)	303 (18.5)	126 (15.6)
3-4	458 (38.7)	588 (40.2)	369 (38.8)	596 (39.9)	842 (39.7)	204 (38.9)	654 (40.0)	311 (38.4)
5-9	397 (33.6)	403 (27.5)	309 (32.5)	413 (27.6)	649 (30.6)	151 (28.8)	477 (29.2)	245 (30.2)
**Child-Pugh score (%)**								
Class A (score 5-6)	801 (67.8)	946 (64.7)	641 (67.4)	966 (64.6)	1419 (66.9)	328 (62.6)	1085 (66.3)	522 (64.4)
Class B (score 7-9)	367 (31.0)	497 (34.0)	300 (31.5)	509 (34.0)	674 (31.8)	190 (36.3)	530 (32.4)	279 (34.4)
Class C (score 10-15)	14 (1.2)	20 (1.4)	10 (1.1)	20 (1.3)	28 (1.3)	6 (1.1)	21 (1.3)	9 (1.1)
**FIB-4 score**								
< 1.45	611 (51.7)	802 (54.8)	507 (53.3)	813 (54.4)	1142 (53.8)	271 (51.7)	912 (55.7)	408 (50.4)
1.45-3.25	483 (40.9)	547 (37.4)	384 (40.4)	565 (37.8)	824 (38.8)	206 (39.3)	616 (37.7)	333 (41.1)
>3.25	88 (7.4)	114 (7.8)	60 (6.3)	117 (7.8)	155 (7.3)	47 (9.0)	108 (6.6)	69 (8.5)
**Warfarin time in therapeutic range (TTR)**								
TTR > 60%	68 (39.5)	86 (27.7)	56 (44.8)	88 (26.8)	132 (35.0)	22 (21.0)	108 (36.6)	36 (22.8)

Table S7. Summary data for outcomes of interests pertaining to Table 4 (stroke and bleeding) in patients without chronic liver disease.

Outcome	No stroke	Stroke	Strata	Drug type
**Time not taking medication (%)**				
n	75102	15467	All patients	Anticoagulants
< 1 week	17730 (23.6)	3919 (25.3)	All patients	Anticoagulants
1 week to 1 month	3492 (4.6)	769 (5.0)	All patients	Anticoagulants
1 to 3 months	2492 (3.3)	526 (3.4)	All patients	Anticoagulants
3 to 6 months	17039 (22.7)	4106 (26.5)	All patients	Anticoagulants
> 6 months	34349 (45.7)	6147 (39.7)	All patients	Anticoagulants
n	12312	974	CHA^{2}DS^2 VASc score 0-1	Anticoagulants
< 1 week	2540 (20.6)	198 (20.3)	CHA^{2}DS^2 VASc score 0-1	Anticoagulants
1 week to 1 month	534 (4.3)	55 (5.6)	CHA^{2}DS^2 VASc score 0-1	Anticoagulants
1 to 3 months	357 (2.9)	32 (3.3)	CHA^{2}DS^2 VASc score 0-1	Anticoagulants
3 to 6 months	2842 (23.1)	279 (28.6)	CHA^{2}DS^2 VASc score 0-1	Anticoagulants
> 6 months	6039 (49.0)	410 (42.1)	CHA^{2}DS^2 VASc score 0-1	Anticoagulants
n	13008	1592	CHA^{2}DS^2 VASc score 2	Anticoagulants
< 1 week	3231 (24.8)	379 (23.8)	CHA^{2}DS^2 VASc score 2	Anticoagulants
1 week to 1 month	690 (5.3)	81 (5.1)	CHA^{2}DS^2 VASc score 2	Anticoagulants
1 to 3 months	436 (3.4)	49 (3.1)	CHA^{2}DS^2 VASc score 2	Anticoagulants
3 to 6 months	3002 (23.1)	479 (30.1)	CHA^{2}DS^2 VASc score 2	Anticoagulants
> 6 months	5649 (43.4)	604 (37.9)	CHA^{2}DS^2 VASc score 2	Anticoagulants
n	32081	5644	CHA^{2}DS^2 VASc score 3-4	Anticoagulants
< 1 week	7980 (24.9)	1519 (26.9)	CHA^{2}DS^2 VASc score 3-4	Anticoagulants
1 week to 1 month	1589 (5.0)	301 (5.3)	CHA^{2}DS^2 VASc score 3-4	Anticoagulants
1 to 3 months	1132 (3.5)	207 (3.7)	CHA^{2}DS^2 VASc score 3-4	Anticoagulants
3 to 6 months	7291 (22.7)	1504 (26.6)	CHA^{2}DS^2 VASc score 3-4	Anticoagulants
> 6 months	14089 (43.9)	2113 (37.4)	CHA^{2}DS^2 VASc score 3-4	Anticoagulants
n	17701	7257	CHA^{2}DS^2 VASc score 5-9	Anticoagulants
< 1 week	3979 (22.5)	1823 (25.1)	CHA^{2}DS^2 VASc score 5-9	Anticoagulants
1 week to 1 month	679 (3.8)	332 (4.6)	CHA^{2}DS^2 VASc score 5-9	Anticoagulants
1 to 3 months	567 (3.2)	238 (3.3)	CHA^{2}DS^2 VASc score 5-9	Anticoagulants
3 to 6 months	3904 (22.1)	1844 (25.4)	CHA^{2}DS^2 VASc score 5-9	Anticoagulants
> 6 months	8572 (48.4)	3020 (41.6)	CHA^{2}DS^2 VASc score 5-9	Anticoagulants
n	170009	53145	All patients	Antiplatelets
< 1 week	50711 (29.8)	16212 (30.5)	All patients	Antiplatelets
1 week to 1 month	9141 (5.4)	2648 (5.0)	All patients	Antiplatelets
1 to 3 months	5336 (3.1)	1615 (3.0)	All patients	Antiplatelets
3 to 6 months	40619 (23.9)	13602 (25.6)	All patients	Antiplatelets
> 6 months	64202 (37.8)	19068 (35.9)	All patients	Antiplatelets
**Outcome**	**No bleeding**	**Bleeding**	**Strata**	**Drug type**
	72721 (80.3)	17848 (19.7)	All patients	Anticoagulants
	179651 (80.5)	43503 (19.5)	All patients	Antiplatelets

Table S8. Summary data for outcomes of interests pertaining to Table 5 (comparison of stroke and bleeding risk between patients with and without chronic liver disease (CLD)).

Strata	No stroke	Stroke	Drug type
**n**	75644	15553	Anticoagulants
**Without CLD**	75102 (99.3)	15467 (99.4)	Anticoagulants
**With CLD**	542 (0.7)	86 (0.6)	Anticoagulants
**Time not taking medication (%)**			
< 1 week	17822 (23.6)	3941 (25.3)	Anticoagulants
1 week to 1 month	3513 (4.6)	772 (5.0)	Anticoagulants
1 to 3 months	2504 (3.3)	528 (3.4)	Anticoagulants
3 to 6 months	17126 (22.6)	4120 (26.5)	Anticoagulants
> 6 months	34679 (45.8)	6192 (39.8)	Anticoagulants
**n**	171467	53505	Antiplatelets
**Without CLD**	170009 (99.1)	53145 (99.3)	Antiplatelets
**With CLD**	1458 (0.9)	360 (0.7)	Antiplatelets
**Time not taking medication (%)**			
< 1 week	51030 (29.8)	16306 (30.5)	Antiplatelets
1 week to 1 month	9196 (5.4)	2663 (5.0)	Antiplatelets
1 to 3 months	5386 (3.1)	1624 (3.0)	Antiplatelets
3 to 6 months	40904 (23.9)	13682 (25.6)	Antiplatelets
> 6 months	64951 (37.9)	19230 (35.9)	Antiplatelets
**Strata**	**No bleeding**	**Bleeding**	**Drug type**
**n**	73234	17963	Anticoagulants
**Without CLD**	72721 (99.3)	17848 (99.4)	Anticoagulants
**With CLD**	513 (0.7)	115 (0.6)	Anticoagulants
**Patients who were adherent (defined as PDC > 80%)**	22010 (30.1)	4813 (26.8)	Anticoagulants
**n**	181047	43925	Antiplatelets
**Without CLD**	179651 (99.2)	43503 (99.0)	Antiplatelets
**With CLD**	1396 (0.8)	422 (1.0)	Antiplatelets
**Patients who were adherent (defined as PDC > 80%)**	63691 (35.2)	13886 (31.6)	Antiplatelets
